# The Dynamics of Plasma Membrane, Metabolism and Respiration (PM-M-R) in *Penicillium ochrochloron* CBS 123824 in Response to Different Nutrient Limitations—A Multi-level Approach to Study Organic Acid Excretion in Filamentous Fungi

**DOI:** 10.3389/fmicb.2017.02475

**Published:** 2017-12-12

**Authors:** Pamela Vrabl, Christoph W. Schinagl, Desirée J. Artmann, Anja Krüger, Markus Ganzera, Ansgar Pötsch, Wolfgang Burgstaller

**Affiliations:** ^1^Institute of Microbiology, University of Innsbruck, Innsbruck, Austria; ^2^Institute of Pharmacy/Pharmacognosy, University of Innsbruck, Innsbruck, Austria; ^3^Plant Biochemistry, Ruhr University Bochum, Bochum, Germany; ^4^School of Biomedical and Healthcare Sciences, Plymouth University, Plymouth, United Kingdom

**Keywords:** *Penicillium ochrochloron*, organic acid excretion, energy charge, catabolic reduction charge, anabolic reduction charge, plasma membrane H^+^-ATPase, alternative oxidase

## Abstract

Filamentous fungi are important cell factories. In contrast, we do not understand well even basic physiological behavior in these organisms. This includes the widespread phenomenon of organic acid excretion. One strong hurdle to fully exploit the metabolic capacity of these organisms is the enormous, highly environment sensitive phenotypic plasticity. In this work we explored organic acid excretion in *Penicillium ochrochloron* from a new point of view by simultaneously investigating three essential metabolic levels: the plasma membrane H^+^-ATPase (PM); energy metabolism, in particular adenine and pyridine nucleotides (M); and respiration, in particular the alternative oxidase (R). This was done in strictly standardized chemostat culture with different nutrient limitations (glucose, ammonium, nitrate, and phosphate). These different nutrient limitations led to various quantitative phenotypes (as represented by organic acid excretion, oxygen consumption, glucose consumption, and biomass formation). Glucose-limited grown mycelia were used as the reference point (very low organic acid excretion). Both ammonium and phosphate grown mycelia showed increased organic acid excretion, although the patterns of excreted acids were different. In ammonium-limited grown mycelia amount and activity of the plasma membrane H^+^-ATPase was increased, nucleotide concentrations were decreased, energy charge (EC) and catabolic reduction charge (CRC) were unchanged and alternative respiration was present but not quantifiable. In phosphate-limited grown mycelia (no data on the H^+^-ATPase) nucleotide concentrations were still lower, EC was slightly decreased, CRC was distinctly decreased and alternative respiration was present and quantifiable. Main conclusions are: (i) the phenotypic plasticity of filamentous fungi demands adaptation of sample preparation and analytical methods at the phenotype level; (ii) each nutrient condition is unique and its metabolic situation must be considered separately; (iii) organic acid excretion is inversely related to nucleotide concentration (but not EC); (iv) excretion of organic acids is the outcome of a simultaneous adjustment of several metabolic levels to nutrient conditions.

## Introduction

The global demand for organic acids and their derivatives as source for chemical building blocks (Sauer et al., 2008; Steiger et al., 2013) and as additives in food and cosmetics industry are on a continuous rise. Albeit filamentous fungi are biotechnologically widely used to meet this market, their ability to produce these metabolites is far from being exploited. One reason is a lack of understanding of even basic physiological aspects of these organisms (e. g., growth development, metabolism, and gene expression), which have been studied comparably less than in bacteria or yeasts (Meyer et al., 2016).

For example, the reason why filamentous fungi excrete organic acids at all is still debated controversially (Neijssel et al., 1993; Ruijter et al., 2002; Plassard and Fransson, 2009; Garcia and Torres, 2011; Vrabl et al., 2012; Knuf et al., 2013) and includes hypotheses like overflow metabolism (Tempest and Neijssel, 1992), charge balance (Slayman et al., 1990), aggressive acidification (Andersen et al., 2009) and some others like energy spilling (Russell, 2007). For most organic acids—if not all—it is still unclear what exactly triggers or impedes their production and excretion (Krull et al., 2010).

However, concepts of systems biology brought the increasing awareness that understanding organisms and their physiology needs to go a few steps further than to search for a single decisive factor in the cause and effect relationship, which is—due to the high complexity of cellular metabolism—rather unlikely (Chubukov et al., 2014). Indeed, systems biology highlighted the need for a quantitative analysis of dynamic interactions between the components of a physiological network. Unfortunately, this is specially difficult with filamentous fungi, because of their enormous, highly environment-sensitive phenotypic plasticity (Foster, 1949; McGetrick and Bull, 1979; Bridge et al., 1987; Rayner, 1996; Avery, 2005; Vrabl et al., 2008; Slepecky and Starmer, 2009; Braaksma et al., 2011; Wösten et al., 2013; Hewitt et al., 2016). This strong phenotypic plasticity of filamentous fungi poses high demands on the standardization of cultivation conditions and the characterization of physiological states and growth phases in submerged culture. Thus, to be successful, an unusual high degree of standardization is necessary.

**Figure 1:**
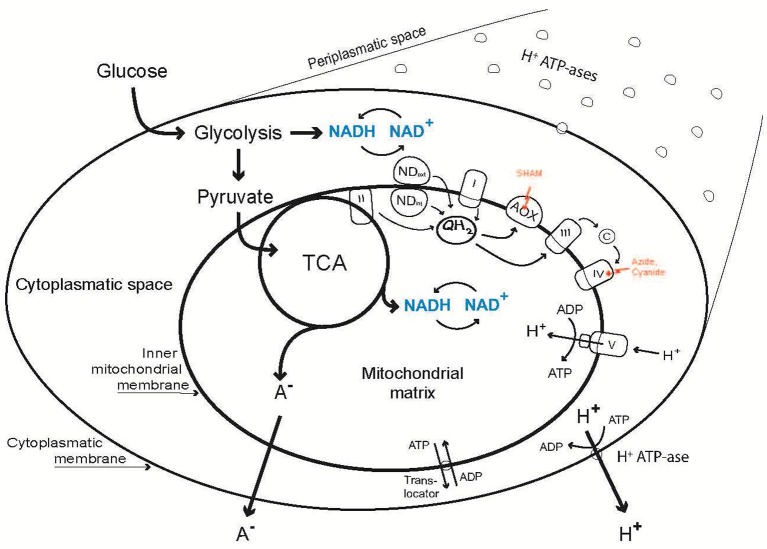
Outline of the relations between organic acid excretion (A^−^) and three levels of cellular energy metabolism: Metabolism (Energy Charge, (ATP + ½ ADP)/(AMP + ADP + ATP); Catabolic Reduction Charge, NADH/(NAD^+^ + NADH); concentration of adenine and pyridine nucleotides), alternative respiration via AOX and the plasma membrane H^+^-ATPase.

In this work we aimed at exploring organic acid excretion in a filamentous fungus—*Penicillium ochrochloron*—from a new point of view by investigating three essential metabolic levels simultaneously. The targeted levels were: (i) the level of the plasma membrane (PM); (ii) the level of (energy) metabolism (M); and (iii) the level of respiration (R). As all three levels are intertwined and connected via intermediary metabolites and energetics (Figure 1), a change in one level will cause consequences on the other levels. This means, that a change in plasma membrane composition and/or the activity of important proteins (e. g., plasma membrane H^+^-ATPase or nutrient transporters) has significant impact on the subsequent catabolic and anabolic fluxes as well as on the energetic state of the hyphae. The respiratory chain is physically connected with the tricarboxylic cycle via the succinate dehydrogenase and indirectly via the NADH turnover. A main consumer of the ATP produced by oxidative phosphorylation is the plasma membrane ATPase, which in turn provides the cell with a proton motive force for nutrient uptake. Last but not least, all excreted metabolites have to pass the plasma membrane.

Of course, in the past all three target levels (PM-M-R) have been connected in one way or another with organic acid excretion in filamentous fungi: (i) plasma membrane, PM (Burgstaller et al., 1997; Jernejc and Legisa, 2001; Roberts et al., 2011), (ii) energy metabolism, M (Kubicek et al., 1980; Nasution et al., 2006a,b; Vrabl et al., 2009) and (iii) (alternative) respiration, R (Kubicek et al., 1980; Kirimura et al., 2000; Gallmetzer and Burgstaller, 2002). So far, these levels have not been explored simultaneously, quantitatively, related to each other and in chemostat culture. Additionally, it is unknown, if and how organic acid excretion and its relation to the PM-M-R dynamics shifts in dependence of the nutrient limitation. Unfortunately, systemic approaches as these are highly challenging. They require not only an extensive standardization of cultivation methods to ensure comparability between different physiological states, but also the application of different sophisticated experimental methods adjusted for each targeted level. Therefore, in this study the PM-M-R dynamics were explored in rigorously standardized chemostat cultivations (Bull, 2010) under energetically different conditions (i. e., glucose, ammonium or nitrate, and phosphate limitation) which vary in the degree of organic acid excretion.

This elaborate task was only possible because we could rely on three decades of experimental experience with our model organism *P. ochrochloron* for which we had the necessary broad range of established methods at our disposal. The resulting data not only provides a unique new point of view for organic acid excretion in filamentous fungi but also highlight the need for the scientific community to focus more on the phenomenon of phenotypic plasticity in these organisms. Especially, as it is connected inevitably with many aspects of experimental methodology and thus the quality of the gained data sets.

## Materials and methods

### Organism and cultivation conditions

*Penicillium ochrochloron* CBS 123824 has developed from a wild type isolate (CBS 123823) after 20 years of passaging on rye. The wild type strain originated from a soil which was strongly contaminated with heavy metals and was initially identified as the closely related species *Penicillium simplicissimum* (Franz et al., 1991). For both strains a re-identification revealed *P. ochrochloron* as the correct species (Vrabl et al., 2008).

The media as well as detailed procedures for pre-culture and chemostat cultivations are described in Schinagl et al. (2016) and Vrabl et al. (2016). In addition, an overview for growth media is given in the supplementary information, section growth media and chemicals. In chemostat cultivations the limiting nutrients were (mM in reservoir): glucose 20, ammonium 2 or 4, phosphate 0.11, and nitrate 2. For chemostat cultivations the following bioreactors were used (Schinagl et al., 2016; Vrabl et al., 2016): Biostat A (Sartorius, Göttingen, Germany), Biostat M (Braun, Melsungen, Germany), KLF 2000 (Bioengineering, Switzerland), all with a working volume of 1.8 L (including 100 mL of pre-culture).

During continuous operation the volume of the culture broth was held constant either by overflow via a short lateral inclined glass pipe at the liquid/air boundary (Biostat M), or via an outlet in the bottom plate of the bioreactor connected to a pump controlled by a balance (KLF 2000), or via a short glass pipe at the side of the bioreactor connected to a pump controlled by a level sensor (Biostat A).

The medium components (in total 1.7 L) were sterilized separately (Schinagl et al., 2016; Vrabl et al., 2016). After blending the medium components and transfer into the bioreactor under sterile conditions, the medium was inoculated with 100 mL of pre-culture (72 h, ammonium exhausted at c. 48 h and glucose excess). Two hours after the inoculation the feed pump was started. Cultures reached a steady state after four hydraulic residence times. Growth proceeded at pH 7 and μ = 0.1 h^−1^. Analytics of main nutrients (glucose, ammonium, and phosphate) as well as determination of biomass and extracellular organic acids were done as specified in Vrabl et al. (2012). Nitrate was determined according to Doblander and Lackner (1996).

### Energy metabolism (M)

#### Sample processing for intracellular nucleoside and nucleotide analysis

Quenching in cold aqueous methanol (−40°C, 60% v/v), the subsequent cold filtration and resuspension in the hot extraction solvent using the FiltRes-device were performed as described in Vrabl et al. (2016). To improve the stability of acid labile nucleotides such as NADH, we modified our formerly used extraction solvent (50% ethanol, Ganzera et al., 2006) and tested the effect of different buffers. We aimed at keeping the pH at above 7.5 throughout the whole sample processing as recommended by Bergmeyer (1974), which is also beneficial for the stability of adenine nucleotides. Ethanolic solutions buffered with HEPES at pH 7.5 are often used in this context (Gonzalez et al., 1997; Lange et al., 2001). However, the shift in pH caused by the elevated extraction temperatures was so far neglected. For HEPES buffers the shift of Δ pH/Δ T (Units per°C) is −0.015 (Dawson et al., 1986), which means that the pH of a extraction solution with pH 7.5 adjusted at 20°C will drop considerably below pH 7 when the extraction solvent is preheated to the extraction temperature of 90°C. In consequence we pursued the following strategy in choosing the appropriate buffer additive: First, the buffer range should guarantee that the pH at extraction conditions is 7.5 or above, which is more easily achieved with buffers with a low temperature dependent shift in pH. Second, the buffer should not negatively interfere with the analytical assay or the HPLC column in any way. Although borate, TAPS and CAPS buffers would meet at least the first criteria, preliminary experiments demonstrated that they did not meet the second criteria (unpublished results). The finally chosen buffer additives were CHES and EPPS (both with a Δ pH/Δ T of −0.011; (Dawson et al., 1986); 10 mM final concentration in the ethanolic extraction solvent). To ensure that the pH stayed above 7.5 during the entire extraction, the initial pH at 20°C was set to 8.6. Method validation was performed for both buffered ethanolic extraction solvents and the unbuffered ethanolic extraction solvent as described in the section “Nucleoside and nucleotide analytics” below. Further sample processing, e. g. sample extraction in a water bath preheated to 90°C, sample evaporation, storage until further analysis and preparation directly before the analysis, followed the protocol described in Vrabl et al. (2016).

#### Nucleoside and nucleotide analysis

As part of an extensive rework of our whole sample processing, also the former analytical method (Ganzera et al., 2006) was adapted to the current needs. To determine the intracellular nucleoside and nucleotide content, a new analytical method was established. All samples were measured with two independent analytical procedures, which were HPLC and CE.

HPLC experiments were performed on a Merck Hitachi EliteChrom HPLC system. The optimum separation could be achieved on a Phenomenex Luna 5 μ C8 (2), 100 Å column (150 × 4.60 mm, 5 μm; Torrance, CA, USA), using a mobile phase comprising 5 mM Dibutylamin (DBA) buffer at pH 6.8 (A) and acetonitrile (B). For elution the gradient started with 99.5% A/0.5% B for 8 min followed by decreasing A in 7 min to 95%, in another 5 min to 90%, to finally reach 65% A in further 10 min. After each run the column was rinsed for 5 min with 70% B and 30% of 0.5% phosphoric acid followed by a 10 min re-equilibration phase (99.5% A, 0.5% B) before the next injection. The wavelength for diode array detection (DAD) was set to 254 nm, the excitation and emission wavelengths for fluorescent detection (FLD) were set to 340 nm and 465 nm, respectively. The separation was performed at 16°C, the flow rate adjusted to 0.8 mL/min. and the injected sample volume was 10 μl.

Capillary electrophoresis (CE) experiments were done using a 3D-CE system from Agilent (Waldbronn, Germany). The capillary used was a fused silica capillary with an inner diameter of 50 μm and an effective length of 62 m (total length 70 cm), which was purchased from Polymicro Technologies (Phoenix, AZ, USA). For the separation of the analytes within 15 min the optimum buffer was 60 mM citric acid and 0.8 mM Cetyltrimethylammoniumbromide (CTAB) in water set to a pH of 4.2 with γ-aminobutyric acid (GABA). Voltage was set to −25 kV, at a temperature of 15°C and Diode-array-detection (DAD) wavelength was 254 nm. The injection was done hydro dynamically at 35 mbar for 6 s. Before each run the capillary was re-conditioned with 0.1 N NaOH, water and running buffer for 3 min each. All required solutions were membrane filtered and replaced periodically. Details on method validation (e. g., linearity, accuracy, precision, repeatability, limit of detection (LOD), and limit of quantitation (LOQ) and chromatograms/electropherograms can be found in the Supplementary Information, section metabolism.

### Respiration (R)

Sample preparation for high resolution respirometry was done as described in Schinagl et al. (2016). Modifications of this respirometric assay concerning (i) the time regime, and (ii) the applied inhibitors and uncouplers were mandatory, because mycelium of *P. ochrochloron* grown with different nutrient limitations displayed not only different physiological properties but also slightly varying morphologies and thus rheological behavior during cultivation. Phosphate-limited mycelium started to clog the overflow pipe between 24 and 48 h of steady state cultivation, which limited the time for sampling and therefore the time regime for the respirometric assay was shortened accordingly, i. e., all three measurements were performed within the first 12 h after reaching steady state conditions.

The inhibitors for studying differently limited steady state mycelia (μ = 0.1 h^−1^) were tested in advance because the phenotypic plasticity of this organism hindered an overall application of one particular inhibitor for all growth limitations. For example, cyanide was clearly the better choice for inhibiting complex IV of glucose-limited grown mycelia (Schinagl et al., 2016) but was not suitable for phosphate-limited grown or for nitrogen-limited grown mycelia at all. Immediately after injection of cyanide to the hyphae in the respirometer chamber a strong oxidative burst was observable, which was also noticed if cyanide was added to the respiration medium without biomass. On the other hand, azide exerted a complete inhibition of complex iV in these mycelia, which was not the case for glucose-limited mycelium as mentioned before. Also the uncoupling agents for the mitochondrial proton gradient were tested for applicability, e. g., FCCP, CCCP, and DNP failed to deliver reproducible results with ammonium-limited grown mycelia.

### Vanadate sensitive plasma membrane H^+^-ATPase activity in membrane fractions (PM)

The principal method was adapted to *P. ochrochloron* by Müller et al. (2001). Here an outline of the method is given. For further details concerning optimizations, assay set up and conditions, as well as the composition of reagents see supplementary information, section plasma membrane. Because this part is less well-documented than the sections Energy Metabolism (Vrabl et al., 2016) and Respiration (Schinagl et al., 2016) the method is described in more detail.

#### Harvest of the biomass

The culture broth from the bioreactor was poured into a 2 L graduated beaker and the volume noted. The broth was transferred into a 5 L metal vessel immersed into ice and within 15 min the temperature of the culture broth was brought to 4°C with an immersion cooler (FT 200, Julabo). The cold fermentation broth was filtered through a cotton cloth and the filtrate checked for bacterial infection. The biomass was washed with 5 L of 4°C deionized water, wrung out, wrapped into tinfoil, and stored at −20°C. An aliquot was dried overnight at 105°C to determine the dry weight.

#### Disintegration of hyphae

In a previous study (Walder, 2011) it was confirmed that freezing at −20°C had no adverse effect on the vanadate sensitive ATPase activity (see Supplementary Information, section Plasma Membrane). Frozen mycelium was defrosted by storage at 4°C overnight, then 25 g of wet weight (= approximately 2 g of dry weight) were suspended in 150 mL of 4°C cold homogenization medium (HM; supplementary information, section plasma membrane). A precooled 350 mL chamber of the glass bead mill Bead Beater (Biospec Products, Bartlesville, USA), containing 200 g of precooled 0.5 mm glass beads, was filled with the mycelial suspension. Air bubbles were removed by gentle stirring with a glass rod and the chamber was filled with homogenization medium to the brim. The chamber was closed and inserted into an ice water jacket filled with 96% (v/v) ethanol precooled to at least −50°C with dry ice. The hyphae were disintegrated for 2 min at 20,000 rpm. After disintegration the temperature of the homogenate was between 0°C and 5°C. The homogenate was then filtered through a nylon net (mesh 100 μm), the volume of the crude extract was determined, and two aliquots were stored at −20°C.

#### Differential centrifugation of the crude extract

Three centrifugation steps were carried out as specified in Müller et al. (2001). The pellet from the third centrifugation was designated as “microsomal fraction” (MF). The main part of the MF was re-suspended by means of an all glass Potter-Elvehjem homogenizer in resuspension medium 1 (RM 1) for further treatment with the aqueous polymer two phase system, an aliquot was re-suspended in resuspension medium 2 (RM 2) for determination of protein and vanadate sensitive ATPase activity. Subcellular fractions were stored at −80°C.

#### Plasma membrane purification from a microsomal fraction

Plasma membranes were purified from a microsomal fraction by aqueous polymer two phase partitioning as optimized by Müller et al. (2001) for mycelia of *P. ochrochloron* from a bioreactor batch culture in a sucrose tartrate medium without pH regulation. For the tests, changes and improvements made in the meantime to this method see supplementary information, section plasma membrane. Here only the final procedure is reported. A general description of the method can be found in Larsson and Widell (2000).

A two phase system (27 g) necessary for a 36 g partitioning system (Larsson and Widell, 2000) was prepared to give final concentrations of 6.1% (w/w) dextran T 500 (lower phase) and 6.1% (w/w) polyethylene glycol 3350 (upper phase). The concentration of the dextran stock solution was checked twice with two different polarimeters. In addition, a washing two phase system of 150 g was also prepared and both phases stored separately at 4°C. The 27 g two phase system (in a 50 mL polycarbonate centrifugation tube; tube 1) was loaded with microsomal fraction (approximately 20 mg of protein; 72 mg of protein at maximum) and completed with RM 1 to a total weight of 6 g. The separation system (tube 1) was incubated on ice for 5 min and then mixed by 40 inversions of the tube by hand. The phases were separated by centrifugation (5 min, 1,500 g, 4°C; HB 6 swinging bucket rotor; Sorvall RC-5B superspeed centrifuge).

To increase yield and purity of the plasma membranes two further phase partitioning steps were applied (for an illustration of the procedure see Figure 1 of Larsson and Widell, 2000).

The lower phase from tube 1 was diluted 1:10, the two pooled upper phases 1:5 with RM 1. Membranes were spun down at 10,000 g for 1 h. Both pellets were re-suspended in RM 2 (composition designed to match the H^+^-ATPase assay) by means of an all glass Potter-Elvehjem homogenizer and stored at −80°C.

#### Analytical methods

Compared to Müller et al. (2001) the analytical methods for protein determination and vanadate sensitive ATPase activity were tested further, improved and partly changed. Here only the final methods are given.

Protein was determined with Serva Blue G based on the dye Coomassie Blue according to Read and Northcote (1981). The assay was carried out either in polystyrene semi-micro cuvettes or in polystyrene flat bottom microplates. Absorption at 595 nm was measured either with a U 2001 spectrophotometer (Hitachi, Tokyo Japan) or a Sunrise microplate reader (Tecan, Männedorf, Switzerland).

Whereas the conditions for the enzyme assay were basically the same as in Müller et al. (2001; see Supplementary Information, section plasma membrane), the method for the quantification of phosphate liberated from ATP was changed. For phosphate determination we now used a modified method of Lanzetta et al. (1979) which was adapted to microplates. The main reason for this change was that the method of Lanzetta et al. (1979) minimized the release of non-enzymatic phosphate.

On one microplate six different assays could be performed, each in triplicate, together with five phosphate standards (pipetting regime see supplementary information, section plasma membrane). The different assays were: with and without Triton X 100 (distinguishing between inside out and right side out membrane vesicles), with and without magnesium (distinguishing between enzymatic and non-enzymatic phosphate release, and with and without sodium ortho-vanadate (inhibiting specifically the P type plasma membrane ATPase). In addition, each of these assays was carried out without ATP/without membranes, without ATP/with membranes, with ATP/without membranes and with ATP/with membranes (Supplementary Information).

Membranes were also analyzed by mass spectrometry with special emphasis on the plasma membrane H^+^-ATPase. For each sample c. 50 μg of proteins were suspended in Laemmli sample buffer (12 mM Tris–HCl pH 6.8, 0.4% (w/v) SDS, 0.02% (w/v) bromophenol blue, 0.1 M DTT, 5% (v/v) glycerol), incubated at 60°C for 30 min on a shaker and loaded onto 10% (v/v) polyacrylamide gels containing 0.1% SDS. The gels were run at RT, thereupon proteins were visualized with a coomassie brilliant blue stain (Dyballa and Metzger, 2009). Gel lanes containing the samples were cut into 12 slices and proteins in gel cubes digested overnight at 37°C with c. 60 μL of a 12.5 ng/μL trypsin solution in 25 mM ammonium-bicarbonate, pH 8.6, essentially as described (Schluesener et al., 2005). For Liquid Chromatography Electrospray Ionization-tandem Mass Spectrometry (LC-ESI-MS/MS) analysis a nanoAcquity UHPLC (Waters) coupled to an LTQ-XL Orbitrap (Thermo) system was used as described (Vera et al., 2013), yet a 75 min gradient was employed: 0–5 min 99% solvent A (0.1% formic acid) and 1% solvent B (0.1% formic acid in 80% acetonitrile), 5–10 min 99–90% A, 10–59 min 90%−70% A, 59–60 min 60–1% A, 60–62 min 1% A, 62–75 min 99% A. The linear ion trap and the orbitrap were operated in sequence, i. e. after a full MS scan on the orbitrap at a resolution of 60,000 MS/MS spectra of the ten most intense precursors were detected in the ion trap.

All database searches were performed using SEQUEST algorithm (Eng et al., 1994) and MS Amanda (Dorfer et al., 2014), both embedded in Proteome Discoverer^TM^ (Rev. 1.4) with a database (UniprotKB December 16, 2008—v1) for the evolutionally closely related *Penicillium chrysogenum* containing 12,773 protein entries. Only tryptic peptides with up to two missed cleavages were accepted. No fixed modifications were considered. Oxidation of methionine and Gln -> pyro-Glu at peptide N-termini were permitted as variable modifications. The mass tolerance for precursor ions was set to 10 ppm; the mass tolerance for fragment ions was set to 0.5 atomic mass units. For search result filtering, a peptide False Discovery Rate threshold of 0.01 (*q*-value) according to Percolator was set in Proteome Discoverer, and at least two unique peptides with search result rank 1 were required. For Top 3 Protein Quantification (T3PQ; Silva et al., 2006), the average area of the three unique peptides of a protein with the largest peak area was calculated by Proteome Discoverer and for normalization respective protein areas were divided by the sum of all proteins area in each sample.

### Chemicals

Chemicals used for growth media see Schinagl et al. (2016) and Vrabl et al. (2016) or supplementary information, section growth media and chemicals. Chemicals (including the respective supplier) used for high resolution respirometry are given in Schinagl et al. (2016). All other chemicals can be found in the according sections.

## Results

In this work organic acid excretion and three metabolic levels (energy metabolism, M; respiration, especially alternative respiration, R; plasma membrane H^+^-ATPase, P) were studied in a filamentous fungus (*Penicillium ochrochloron*; Figure 1) simultaneously for the first time. In addition, the PM-M-R dynamics were explored in rigorously standardized chemostat cultures under energetically different conditions, i. e., carbon, nitrogen and phosphate limitation, which vary in the degree of organic acid excretion.

**Figure 2:**
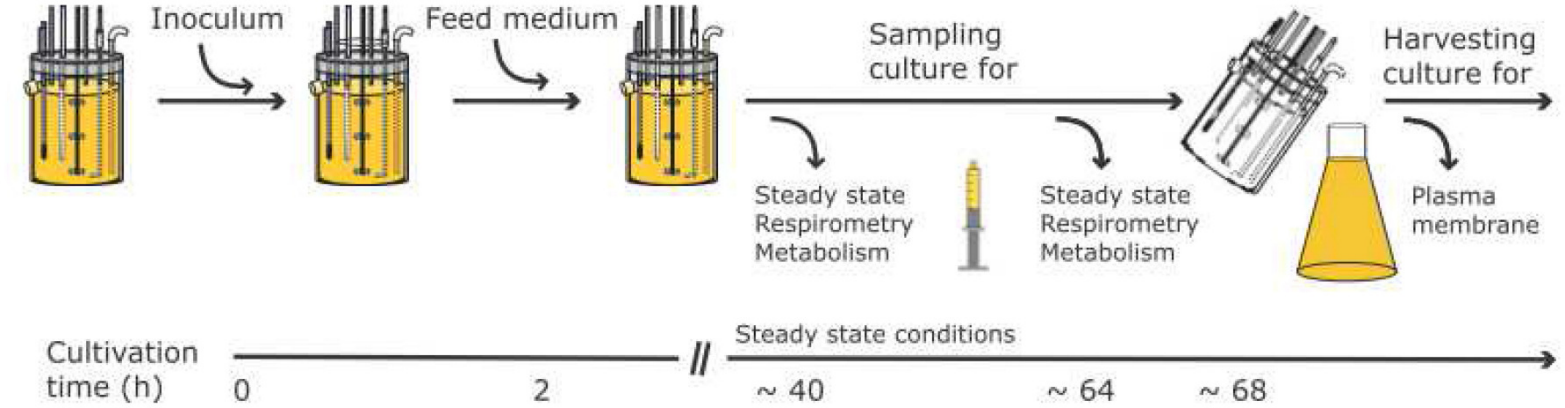
General workflow and timeline for the experiments performed for the three targeted levels (PM-M-R).

The general workflow of the experiments is depicted in Figure 2. During the steady state of a chemostat run samples were taken: (i) to determine biomass and the concentration of main nutrients; (ii) to stop metabolism rapidly with cold methanol for extracting nucleotides; and (iii) to challenge the electron transport system with inhibitors using high resolution respirometry. At the end of cultivation the biomass was harvested and plasma membranes isolated by aqueous polymer two phase partitioning. The plasma membrane H^+^-ATPase was then quantified using an enzymatic assay (total amount as well as specific activity) and mass spectrometry.

### Characterization of growth

The growth of a microorganism, which is to be used for a physiological study, should be characterized thoroughly (Egli, 2015). This concerns media composition and culturing conditions, as well as overall growth parameters such as specific growth rate (μ), oxygen consumption rate (q_O2_), glucose consumption rate (q_Glu_) and biomass concentration. Although these factors are commonly studied in batch culture with the microorganism exhibiting different growth phases and facing ever changing conditions, it is also advisable to investigate them in the steady state of chemostat cultivation.

Following Egli (2015) it was assured that the media used led to a distinct growth limitation by one single nutrient. In Table 1 growth parameters of *P. ochrochloron* during the steady state of chemostat cultivation with four different nutrient limitations are given. A few aspects shall be pointed out. First, ammonium-limited growth was done in the beginning with 4 mM NH_4_^+^ and later with 2 mM NH_4_^+^. The reason for this was that the morphology of ammonium-limited grown mycelia led to the formation of aggregates, especially at the higher concentration of with 4 mM ammonium. This formation of aggregates gave rise to problems with the overflow pipe (biomass enrichment) and the sampling tube (clogging) of the Biostat M bioreactor. Halving the ammonium concentration reduced these problems. In addition, halving the ammonium concentration also halved the biomass as expected according to continuous culture theory (Table 1). Strikingly, the q_O2_ of ammonium-limited grown mycelia was higher with the lower biomass concentration, whereas the q_Glu_ behaved just oppositely. So far we cannot offer any reliable explanation for this opposite behavior.

**Table 1:** Characterization of growth of *Penicillium ochrochloron* in chemostat culture with different limiting nutrients (glucose, ammonium, phosphate, and nitrate).

	**Glucose feed [20 mM] *n* = 3**	**${\text{NH}}_{4}^{+}$ feed [4 mM or 2 mM] *n* = 4 (4 mM) *n* = 2 (2 mM)**	**${\text{PO}}_{4}^{3-}$ feed [0.1 mM] *n* = 2**	**${\text{NO}}_{3}^{-}$ feed [2 mM] *n* = 3**
μ [h^−1^]	0.1	0.1	0.1	0.1
pH	7.0	7.0	7.0	7.0
Duration steady state [h]	48	48	48	48
Steady state concentration of limiting nutrient [μM]	31 ± 9	51 ± 37 (4) 600 ± 474 (2)	0.7 ± 0.3	1.7 ± 1.2
Biomass [(g TG) L^−1^]	3.7 ± 0.4^*^	3.1 ± 0.4 (4) 1.4 ± 0.07 (2)	3.1 ± 0.2	1.0 ± 0.2
q_O2_ [mmol g^−1^ h^−1^]	1.6 ± 0.2	1.1 ± 0.3 (4) 3.1 ± 0.4 (2)	1.4 ± 0.2	1.7 ± 0.3
Technical quotient (TQ) (CO_2_/O_2_)	1.1 ± 0.01	1.4 ± 0.08 (4) 0.95 ± 0.07 (2)	1.2 ± 0.07	0.80 ± 0.02
q_Glu_ [mmol g^−1^ h^−1^]	0.56 ± 0.05	1.3 ± 0.3 (4) 0.97 ± 0.5 (2)	0.80 ± 0.3	1.2 ± 0.8

Bioreactors (Biostat M, Biostat A, Biostat B, KLF 2000) were operated with 1.8 L of medium, and at 30°C, 0.56 vvm, and an impeller tip speed of 2.26 m s^-1^. A steady state (oxygen consumption, carbon dioxide production, biomass concentration) was achieved about 40 h after inoculation.

* *This value is unexpectedly high and results in an unrealistic biomass yield on glucose. The biomass determination was, however, correct. The reason for the high value was probably an accumulation of the biomass due to the construction of the overflow device in the vessel (Gallmetzer and Burgstaller, 2001). We decided to retain this value in the table, because this was the actual biomass respiring and consuming glucose, and it was proofed that the culture was in steady state*.

During the experiments it became more and more obvious that different nutrient limitations led to different morphological/physiological phenotypes. This had consequences for sample preparation methods and analytical methods. Distinct phenotypic differences were observed concerning the filtration of the biomass, the response to metabolite extraction solutions buffered with different buffers, the constancy of the respiration rate in the high resolution respirometer, the aggregation behavior of the biomass during stirring in the chamber of the high resolution respirometer, and the response to various inhibitors of the electron transport system. Because of these effects some experiments could not be carried out to their full extent. For instance, some effects of inhibitors on ammonium-limited grown mycelia could not be quantified in high resolution respirometry because the respiration without inhibitors did not stabilize sufficiently.

### Metabolism (M)

**Figure 3:**
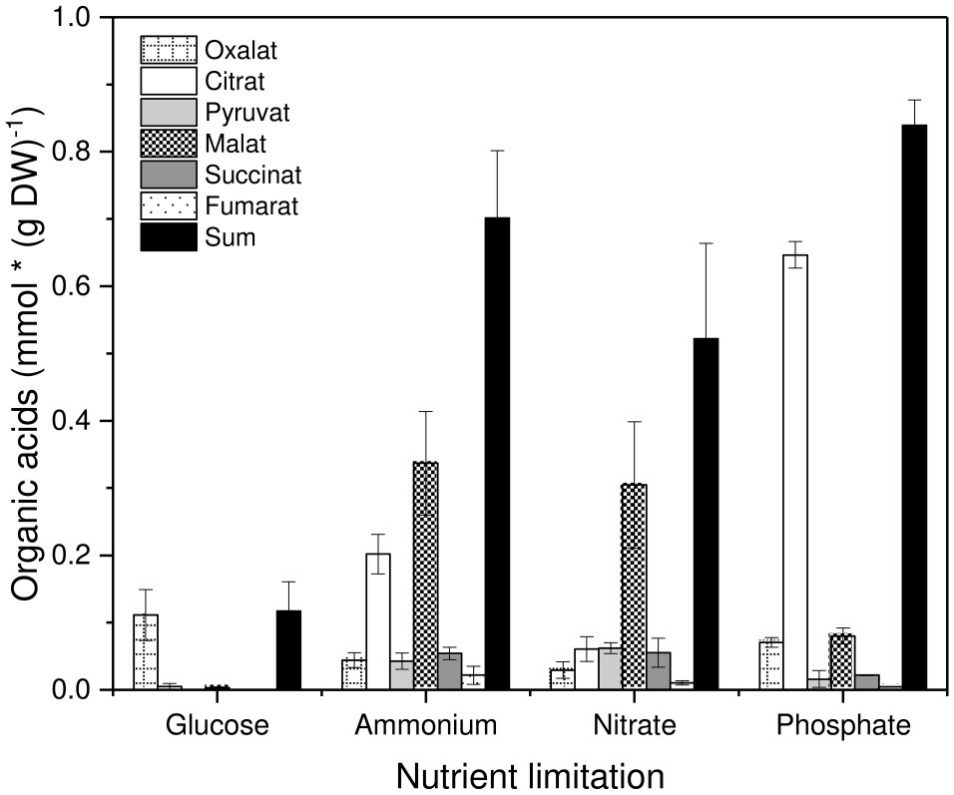
Excretion of organic acids by *Penicillium ochrochloron* CBS 123.824 during growth in chemostat with different limiting nutrients (glucose, ammonium, nitrate, and phosphate). Data for each nutrient limitation were derived from the steady state and represent averages ± standard deviation of *n* = 5 samples from three independent chemostats (glucose and ammonium limitation), *n* = 4 samples from two independent chemostats (nitrate limitation) and *n* = 2 samples from two independent chemostats (phosphate limitation).

Concerning organic acid excretion with different nutrient limitations the following picture emerged (Figure 3). Glucose-limited grown mycelia excreted only the C2 compound oxalate in a significant amount. All other nutrient limitations exhibited a decreased excretion of oxalate and a strongly increased excretion of citrate and malate as well as a slightly increased excretion of pyruvate, succinate and fumarate. In general, organic acid excretion was distinctly increased compared to glucose-limited grown mycelia. These results are in accordance with previous findings concerning this strain of *P. ochrochloron* (Vrabl et al., 2012).

**Figure 4:**
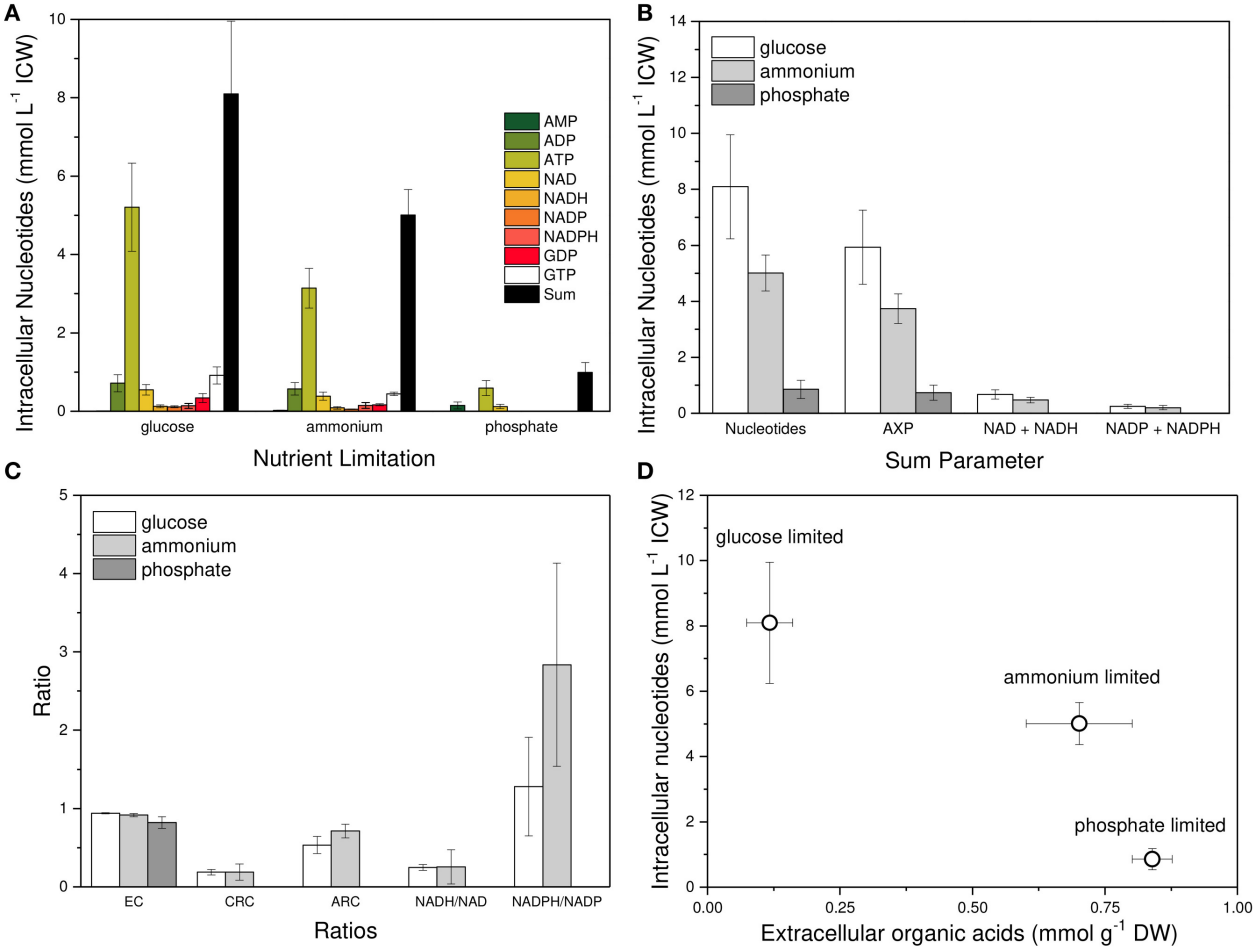
Nucleotide concentrations and their relative relations to each other in *Penicillium ochrochloron* CBS 123.824 during growth in chemostat cultures with different nutrient limitations at μ = 0.1 h^−1^, 30°C and pH 7. Limiting nutrients were glucose (20 mM), ammonium (2 mM), and phosphate (0.1 mM). **(A)** Nucleotide levels in dependence of the nutrient limitation. **(B)** Sum parameters of all targeted nucleotides: adenine nucleotides (AXP), NADH, and NADH, NADP and NADPH, and GDP and GTP (GXP). **(C)** Relative ratios of nucleotides to each other expressed as Energy Charge (EC), Catabolic Reduction Charge (CRC), Anabolic Reduction Charge (ARC), NADH/NAD-ratio and NADPH/NADP-ratio. **(D)** Correlation between organic acid excretion and intracellular nucleotide concentration. Samples for intracellular nucleotide analysis were subjected to an immediate cold methanol stop and further processed using the newly developed FiltRes device (Vrabl et al., 2016). Nucleotides were extracted with hot ethanol and quantified by gradient reversed phase HPLC and CE. For method optimization see supplementary information, section metabolism. Nucleotide data for each nutrient limitation were derived from three subsequent days of steady state cultures and represent averages ± standard deviation of *n* = 14 (glucose limitation), *n* = 13 (ammonium limitation) and *n* = 12 (phosphate limitation) samples.

Figure 4 depicts the intracellular concentration of single adenine and pyridine nucleotides, the sum of these nucleotides, the ratio parameters, and the relation between nucleotide concentrations and organic acid excretion (Krüger, 2013). Three inferences are obvious. First, single nucleotides, as well as the sum of nucleotides, responded strongly to the type of nutrient limitation with glucose limitation showing the highest value (Figures 4A,B). Second, the EC and the CRC were much less dependent on the kind of nutrient limitation (Figure 4C). And third, organic acid excretion was inversely correlated to nucleotide concentration (Figure 4D).

### Respiration (R)

**Figure 5:**
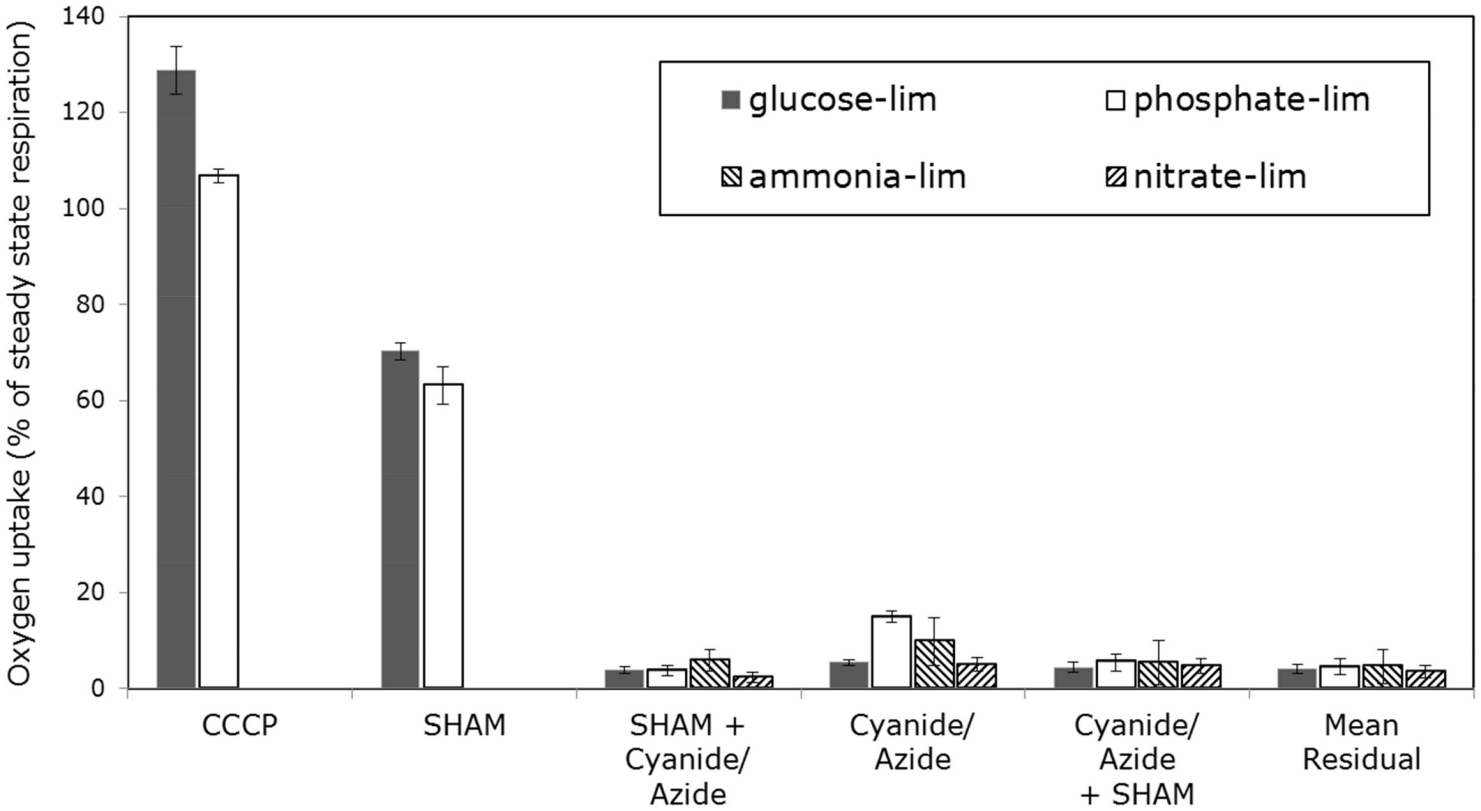
Normalized oxygen consumption rate of *Penicillium ochrochloron* CBS 123.824 mycelia grown in chemostat culture (μ = 0.1^−h^). Oxygen consumption was measured with high resolution respirometry following minimal sample preparation. Limiting nutrients were glucose (20 mM), ammonium (2 mM), nitrate (2 mM) and phosphate (0.1 mM). Intact hyphae were treated with CCCP, Rotenone, Antimycin A, SHAM, Cyanide and Azide. Oxygen consumption rates were normalized to the oxygen consumption rate of untreated mycelia (i.e. 100% value). The oxygen uptake above 100% of CCCP-treated mycelium was regarded as uncoupling capacity. Bars represent averages ± standard deviation of independent chemostat cultures, *n* = 3 (glucose and ammonium limitation) and *n* = 2 (nitrate and phosphate limitation). The data for glucose-limited grown mycelia were already published in (Schinagl et al., 2016).

In Figure 5 the normalized oxygen uptake rates of mycelia grown with different nutrient limitations in response to inhibitors of the ETS shown (the data for glucose-limited grown mycelia were already published in Schinagl et al., 2016). Oxygen uptake was determined by high-resolution respirometry with as little as possible manipulations of the sample taken from the chemostat (Schinagl et al., 2016). Three main conclusions can be drawn: (i) the electron transport system (ETS) of glucose-limited grown mycelia can be uncoupled to a higher degree by the protonophor CCCP than mycelia grown with other nutrient limitations. (ii) Secondly, all mycelia showed an inhibition of oxygen uptake by SHAM, if SHAM was applied as the first inhibitor in a sequence of inhibitors and (iii) only the ETS of phosphate-limited grown mycelia showed a distinct inhibition by SHAM, if SHAM was applied after azide.

Steady state and alternative respiration of these differently limited mycelia showed—due to the phenotypic plasticity—varying accessibility, e. g., for inhibitors of complex IV. Most strikingly, there was no way for both nitrogen-limited mycelia to gain stable respiring hyphae—not even by supplementation of nitrogen—in the high-resolution respirometer although oxygen uptake rates during cultivation in the chemostat were stable. The respiration of both nitrogen-limited grown mycelia was also not stabilizing 15 min after the application of SHAM (inhibitor for AOX), but nevertheless SHAM showed a clear diminishing effect on oxygen uptake. Apart from this qualitative observation on the effect of SHAM, no meaningful quantitative estimation was feasible. Furthermore, an increasing effect of all three uncoupling agents used in this study was not reproducible with ammonium-limited grown mycelia. Interestingly, inhibition of complex I differed substantially between nitrate- and ammonia-limited hyphae. According to the measurements, 50 ± 4% of the oxygen uptake in ammonium-limited hyphae was caused by electrons entering the ETS via complex I. This was in strong contrast to the measured 93 ± 3% of oxygen uptake via complex I in nitrate-limited steady state hyphae. To summarize the findings for nitrogen-limited hyphae, (i) the quantitative estimation of electrons being diverted to AOX and (ii) the uncoupling of ETS was not realizable. The (iii) amount of electrons entering ETS via complex I differed substantially between both sources of nitrogen.

Quantitative conclusions concerning AOX activity are only possible for glucose-limited grown and phosphate-limited grown mycelia, as this parameter could not be quantitated for both nitrogen-limited grown mycelia. Inhibiting AOX with SHAM as the inhibitor added first decreased in both mycelia the initial uptake rate within the same magnitude. This indicated that AOX was constitutively present and active. If complex IV was inhibited first, phosphate-limited grown mycelia showed an activation of AOX with respect to the classical experimental approach (i. e. inhibition of AOX after a complete inhibition of complex IV). No such AOX activity was found in glucose-limited steady state mycelia.

Uncoupling the ETS with CCCP allows insights into the theoretical maximum respiratory capacity of the mycelium. Comparing the maximum respiratory capacity of glucose-limited mycelium and phosphate-limited mycelium to their respective steady state respiration (expressed as 100% value) revealed the following (Figures 5, **7**): In glucose-limited mycelium the maximum respiratory capacity was approximately 29% higher than the steady state respiration, indicating that there is still surplus respiratory capacity to meet metabolic demands. This is in line with a previous study with *P. ochrochloron*, where we found oxygen consumption of glucose-limited mycelium increased after a glucose pulse (Vrabl et al., 2008). In contrast, in phosphate-limited mycelium the maximum respiratory capacity was merely 7% higher than the corresponding steady state respiration. This means that under these conditions phosphate-limited grown mycelia approached the maximum respiratory capacity far more to meet metabolic demands.

The residual oxygen uptake of all four tested nutrient limitations—with inhibitors for AOX and complex IV present—was approximately 5%.

### Plasma membrane

**Figure 6:**
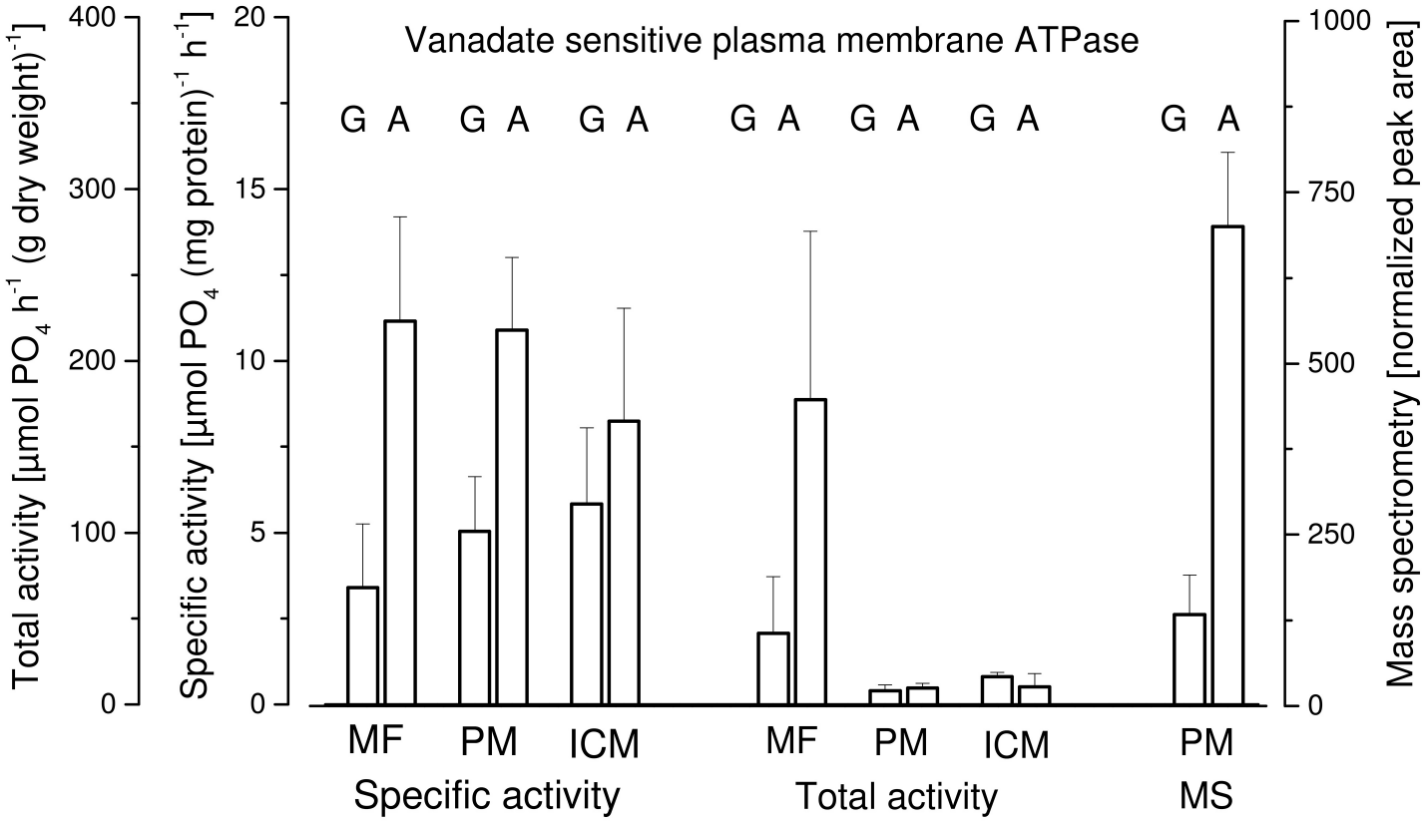
Vanadate sensitive plasma membrane H^+^-ATPase in membrane fractions of *Penicillium ochrochloron* CBS 123.824 grown in chemostat culture with two limiting nutrients (glucose, G; ammonium, A). Plasma membranes (PM) were enriched from a homogenate (glass bead mill) using differential centrifugation yielding a microsomal fraction (MF) which was further subjected to partitioning in an aqueous polymer two phase system. Membranes from both phases of the two phase system (lower phase, intracellular membranes; ICM; upper phase, plasma membranes, PM) were collected and the total, as well as specific vanadate sensitive ATPase activity were determined. Membranes from the upper phase (PM) were also subjected to mass spectrometry and proteins quantified by the T3PQ method (normalized peak area). The values are the average of three (glucose limitation) or four (ammonium limitation) bioreactor runs.

In Figure 6 three parameters concerning the vanadate sensitive ATPase activity in membrane fractions of *P. ochrochloron* are given: the specific vanadate sensitive activity per mg of protein, the total vanadate sensitive activity per gram of dry weight, and the normalized peak area from a mass spectrometric analysis of the membranes (Dengg, 2014; Gasser, 2014). Only two types of mycelia were analyzed: glucose-limited grown and ammonium-limited grown. Three membrane fractions were analyzed: a microsomal fraction (MF; a mixture of plasma membranes and intercellular membranes), and the membranes collected from the upper (PM; should mostly be plasma membranes) and lower phase (ICM; should mostly be intracellular membranes) of the aqueous polymer two phase system. Interpreting specific activity and mass spectrometry data both led to the same conclusion: in ammonium-limited grown mycelia the vanadate sensitive plasma membrane H^+^-ATPase was more abound and more active compared to glucose-limited grown mycelia. Concerning the total activity this picture was confirmed in the microsomal fraction but not for total activity in PM and ICM. The reason for this is unclear.

An additional result of these experiments was that plasma membranes were not predominantly found in the upper phase of the two phase system—as it would be expected—but were distributed between upper and lower phase. This indicated that the two phase system—optimized for a mycelium grown in batch culture with a different medium (Müller et al., 2001)—was not optimal for chemostat mycelia and should be optimized accordingly.

## Discussion

To explore the PM-M-R dynamics of different nutrient limitations means also to deal with various fungal phenotypes. While it is established that methods have to be scrutinized and—if necessary—adapted to each organism anew, the influence of varying phenotypes of one single organism on methodological issues has received little attention so far. As we will show in the first part of this discussion (Phenotypic Plasticity and Physiological Investigations) phenotypic plasticity can have indeed a substantial impact on the applied methods and should be considered when applying comparative systematic approaches to different phenotypes. In the second part (Organic Acid Excretion and the PM-M-R Dynamics) we present scenarios which relate the PM-M-R dynamics and the excretion of organic acid with different nutrient limitations of steady state cultures.

### Phenotypic plasticity and physiological investigations

It is common knowledge that methods cannot be transferred from one species to another without a preceding critical test of their suitability. It is less clear however, if a set of methods—once established for one species—can be applied unexamined to all of its phenotypes. Data from the literature indicated that this might not be the case (da Luz et al., 2014; Zakhartsev et al., 2014; Vrabl et al., 2016) and the findings of this work support this hypothesis too. Using the multilevel approach of this study, we found that merely varying the concentration of one main nutrient with otherwise identical cultivation conditions (i. e., chemostat!), affected all targeted levels in one or another way like the filtration behavior, sample extraction, aggregation behavior or respiration rates. In consequence, some experimental protocols could not be applied for all explored phenotypes without adaptation, or even failed completely. Especially our findings with high resolution respirometry highlight the need to critically examine experimental techniques when being transferred to other phenotypes. For instance, while the established protocol for high resolution respirometry with glucose-limited grown mycelia (Schinagl et al., 2016) could be used with minor modification for a phosphate-limited grown phenotype, this was not possible at all for any nitrogen-limited (ammonium and nitrate) phenotype.

The transfer of ammonium-limited grown mycelia from chemostat conditions to that in the high resolution respirometer changed the physiological state from “energy excess and growing” to “energy excess and non-growing.” This adaptation resulted in a non-constant respiration rate in the high resolution respirometer at least during the recording time of 30 min. For filamentous fungi it is documented that these organisms adapt their physiology rapidly to a decrease in nitrogen availability by lowering the rate of catabolism, amongst others also the rate of respiration (Slayman, 1973; Mason and Righelato, 1976; Kim et al., 1995; Vrabl et al., 2009). Thus, a decreasing respiration rate in the high resolution respirometer would have been unavoidable, because of physiological reasons. We therefore conclude that sample preparation and analytical methods must be carefully tested and adapted not only to each organism, but also for each physiological phenotype.

### Organic acid excretion and the PM-M-R dynamics

#### Experimental premise

To study the interrelation of organic acid excretion and PM-M-R dynamics, several experimental premises had to be made: (i) what kind of nutrient limitations will be used, (ii) what type of cultivation will be applied (e. g., batch vs. chemostat) and (iii) how to tackle the interpretation of the gained results.

**Figure 7:**
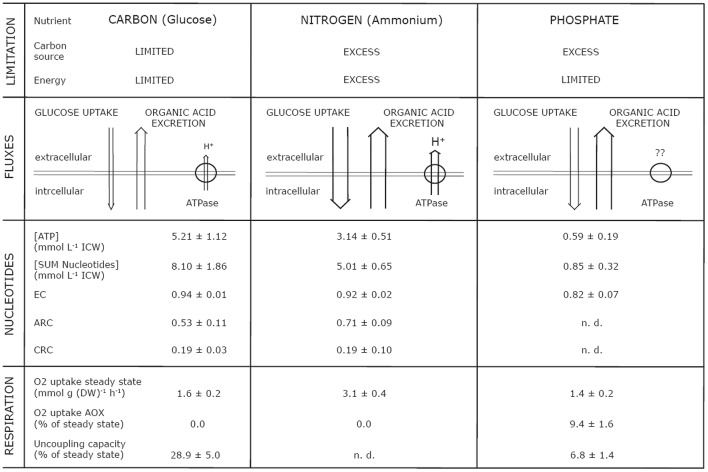
Overview of parameters simultaneously analyzed from plasma membrane, energy metabolism, and respiration in dependence of energetic and nutritional limitations in steady state mycelia (μ = 0.1^−h^) of *Penicillium ochrochloron* CBS 123.824. Abbreviations: EC, Energy Charge; CRC, Catabolic Reduction Charge; ARC, Anabolic Reduction Charge; n. d., not determinable.

First, we chose to explore three different nutritional states (limiting nutrients: glucose, ammonium and nitrate, phosphate), which vastly differ in the availability of energy and carbon (Figure 7). Thus, three distinct physiological states were expected: (i) energy and carbon limitation (C), (ii) energy and carbon excess (N), and (iii) energy limitation and carbon excess (P). It is obvious that N- and P-limitation result in very different physiological conditions for a cell. Nevertheless, both mycelia exhibited an enhanced degree of organic acid excretion (Figure 3). This offered a further opportunity to investigate the phenomenon of organic acid excretion from another perspective, namely high organic acid excretion in a different bioenergetic state.

Second, we exclusively applied chemostat cultivation (Bull, 2010). In this context two remarks are appropriate: (i) one must carefully consider whether the medium and the dilution rate chosen enable growth with a distinct limitation by a single nutrient avoiding an unintendedly established dual limitation (Egli, 1991, 2015). In all conducted chemostat cultivations, distinct single limitations were assured; (ii) furthermore, all chemostat cultivations were performed with the same dilution rate (D = 0.1 h^−1^), meaning that mycelia always grew with the same specific growth rate of μ = 0.1 h^−1^. Therefore, the anabolic energy load (i. e., the energy consumption for growth) must have been rather similar for all mycelia independent of the imposed nutrient limitation. Organic acid excretion could therefore be studied at an equal demand of anabolic energy.

And third, because of the complexity of organisms, simple causal chains may be difficult or even impossible to prove. In addition, a simple and monocausal relation of cause and effect seems improbable because regulation processes in cells are pleiotropic or like a network (Mensonides et al., 2013). We therefore confined ourselves to a phenomenological level, aiming at two directions: (i) to produce well confirmed sets of data; and (ii) to interpret the resulting data from the viewpoint of a potential pleotropic regulation of metabolism.

#### The three single targeted levels in response to different nutrient limitations

##### Energy metabolism

In a previous work with another strain of *P. ochrochloron* (Vrabl et al., 2009) we had to reject the hypothesis of a positive correlation between the concentration of ATP (or EC) and the excretion of organic acids. In the present study, a very clear result was that the increased organic acid excretion by ammonium- and phosphate-limited grown mycelia correlated with a lower intracellular ATP concentration, or with a lower sum of nucleotides, respectively (Figure 4). This was further combined with an increased glucose consumption rate and an either increased (ammonium-limited grown mycelia) or decreased (phosphate-limited grown mycelia) oxygen consumption rate (Table 1). This indicated that increased organic acid excretion occurred at distinctly different physiological states—thus pointing to the fact that drawing a conclusion from considering one factor only (e. g., glucose consumption rate or oxygen uptake rate) might be quite misleading. A well supported conclusion, however, is that a lower ATP concentration allows a higher glycolytic flux (Mensonides et al., 2013).

While there was a strong change in the levels of single nucleotides in dependence of the nutrient limitation, we did not find any significant changes in anabolic reduction charge (ARC), Catabolic Reduction Charge (CRC) or Energy Charge (EC) (Figure 4, Table 2) when comparing glucose and ammonium limitation. Due to the extremely low nucleotide contents of phosphate-limited mycelium, which were often below detection limits, most ratios could not be assessed appropriately for this phenotype. In literature the CRC is often reported to be about 0.03–0.07, whereas the ARC values are typically approximately ten times higher (Stephanopoulos et al., 1998). Thus, CRC values in *P. ochrochloron* were rather high. This seems, however, not to be uncommon for filamentous fungi (Führer et al., 1980; Table 2).

**Table 2:** Synopsis of nucleotide ratios in filamentous fungi characterizing energy metabolism.

**Organism**	**Cultivation**	**Physiological status**	**EC**	**ATP/ADP**	**CRC**	**NADH/NAD^+^**	**ARC**	**NADPH/NADP^+^**	**PNF**	**References**
*P. ochrochloron* CBS 123.824	Chemostat	Glucose-limited	0.94 ± 0.007	7.94 ± 1.157	0.19 ± 0.034	0.25 ± 0.036	0.53 ± 0.108	1.28 ± 0.629	0.37 ± 0.063	Present study
	Chemostat	Ammonium-limited	0.92 ± 0.020	5.77 ± 1.409	0.19 ± 0.104	0.26 ± 0.218	0.71 ± 0.089	2.84 ± 1.296	0.44 ± 0.150	Present study
	Chemostat	Phosphate-limited	0.82 ± 0.075^a^	–^a^	–	–	–	–	–	Present study
*P. ochrochloron* CBS 123.823	Chemostat	Glucose-limited	0.73 ± 0.037	1.79 ± 0.128	–	–	–	–	–	Vrabl et al., 2009
	Chemostat	Ammonium-limited	0.69 ± 0.014	1.61 ± 0.156	–	–	–	–	–	Vrabl et al., 2009
*T. aureoviride*	Chemostat	Glucose-limited	0.90	13.6	–	–	–	–	–	Pitt and Bull, 1982
	Chemostat	Ammonium-limited	0.92	8.15	–	–	–	–	–	Pitt and Bull, 1982
*P. chrysogenum DS17690*	Chemostat	Glucose-limited	0.92	6.3	–	–	–	–	–	Nasution et al., 2006b
*P. chrysogenum*	Bioreactor- batch	Unlimited growth	–	–	0.04 ± 0.01	0.04	0.40 ± 0.05	0.56	0.08 ± 0.02	Führer et al., 1980
*Monascus ruber* ATCC 96218	Bioreactor- batch	Fast growth period	–	–	0.12^b^	0.13–0.14^b^	0.17–0.20^b^	0.20–0.25^b^	0.30 – 0.41^b^	Hajjaj et al., 1998
*A. nigerN400 CBS 120.4*	Unknown	Unknown	–	–	≤ 0.1	–	0.2–0.5	–	–	Witteveen et al., 1994
*A. niger* B60	Bioreactor- batch	Unlimited growth	–	–	0.10 ± 0.02	0.11	0.37 ± 0.05	0.57	0.11 ± 0.02	Führer et al., 1980
	Bioreactor- batch	Citric acid production	–	–	0.14–0.18	0.12–0.18	0.36–0.39	0.58–0.63	0.30–0.38	Führer et al., 1980
*A. niger* NW131	Bioreactor- batch	Late exponential phase (48 h)	0.897	5.51	0.012	0.012	–	–	–	Ruijter and Visser, 1996
*N. crassa* (various strains)	Shake flasks - batch	Mid exponential phase (various media)	–	–	0.35 ± 0.03	0.57 ± 0.043	0.65 ± 0.05	1.87 ± 0.338	0.13 ± 0.024	Brody, 1972^c^
*P. notatum* Westling (IMI 15378)	Bioreactor- batch	Initial vegetative mycelium	0.58 ± 0.05	0.80	0.22 ± 0.02	0.28	0.31 ± 0.04	0.45	0.23 ± 0.02	Mosley et al., 1989^c^
	Bioreactor- batch	8 h vegetative mycelium	0.64 ± 0.05	0.79	0.24 ± 0.03	0.32	0.37 ± 0.05	0.53	0.27 ± 0.05	Mosley et al., 1989^c^
	Bioreactor- batch	8 h Ca^2+^ induced mycelium	0.73 ± 0.02	1.10	0.23 ± 0.06	0.19	0.47 ± 0.20	1.09	0.15 ± 0.04	Mosley et al., 1989^c^

*EC (Energy charge) calculated as ([ATP] + 0.5 [ADP])/([AMP] + [ADP] + [ATP])*.

CRC (catabolic reduction charge) calculated as [NADH]/([NADH] + [NAD^+^]).

*ARC (anabolic reduction charge) calculated as [NADPH]/([NADPH] + [NADP^+^])*.

*PNF (phosphorylated nucleotide fractions) calculated as ([NADP^+^] + [NADPH])/([NAD^+^] + [NADH])*.

a *ADP below detection limit*.

b *Results obtained with different quenching but same extraction methods*.

c *Sampling and metabolic stop possibly too slow for these metabolites*.

##### Respiration

The uncoupling capacity of the respiratory chain differed substantially between glucose- and phosphate-limited cultures (Figure 5). While glucose-limited grown mycelia exhibited a considerable excess capacity of oxygen uptake (Schinagl et al., 2016), the oxygen uptake rates in phosphate-limited grown mycelia were almost at their maximum capacity. One reason for this might be the difference in glycolytic fluxes (Table 1). The high external glucose concentrations (200 mM) forced a high catabolic flux upon the phosphate-limited phenotype which inevitably had consequences on the fluxes through the tricarboxylic cycle and the ETS.

As observed by Schinagl et al. (2016) and others (Lambowitz and Slayman, 1971; Kirimura et al., 1987), the sequence of inhibitors applied determined the extent of obtained AOX activity (Figure 5). Following the classical definition of AOX respiration (i. e., cyanide insensitive and mostly salicylhydroxamic acid sensitive respiration), AOX activity is commonly determined by applying cyanide first, followed by SHAM. With this sequence, only phosphate-limited grown mycelia showed a distinct AOX activity. Reversing the sequence, however, showed a constitutively present AOX activity in all phenotypes (albeit AOX activity could not be determined quantitatively in nitrogen-limited cultures) which points at two important issues. First, alternative respiration in phosphate-limited grown mycelia might play a more central role in this phenotype, because of the very low rate of ATP synthesis. Thus, alternative respiration might have been more relevant to maintain the catabolic flux. Second, this sequence dependency confirms our previous hypothesis (Schinagl et al., 2016) that—like in plants (Sluse and Jarmuszkiewicz, 1998; Siedow and Umbach, 2000)—the electron fluxes to both terminal oxidases, which are connected via the quinone pool, are dynamically adjusted to metabolic demands. Or, in other words, AOX activity most likely does not serve as a safety valve as had been suggested before (Kubicek et al., 1980), but is rather in a constant interdependent dynamic competition for electrons with the cytochrome pathway.

In fungi the residual respiration, which is the remaining oxygen uptake with inhibitors for both terminal oxidases present, is hypothesized to be caused by either leak respiration or non-mitochondrial anabolic oxygen consumption (Rosenfeld and Beauvoit, 2003). In *P. ochrochloron* the residual respiration lies within the same range for all explored phenotypes (Figure 5). The fact that this residual oxygen uptake is unaffected by changes in the phenotype indicates that the involved processes are most likely mirroring a very basic demand for oxygen in the metabolic network.

##### Plasma membrane

Compared to glucose-limited grown mycelia, ammonium-limited grown mycelia contained a higher amount of plasma membrane H^+^-ATPase, which also exhibited a higher specific vanadate sensitive ATPase activity (Figure 6). Unfortunately, limited funds did not allow determination of the plasma membrane H^+^-ATPase in phosphate-limited grown mycelia and we currently can only make assumptions on this issue (see below).

There is little knowledge about the dynamics of changes in the plasma membrane in filamentous fungi due to changes in environmental conditions, and to our knowledge no information is available about the plasma membrane dynamics in relation to energy metabolism and the respiratory chain. However, since the plasma membrane H^+^-ATPase is one of the main ATP consumers within the fungal cell (Sanders, 1988), it is intimately linked with the ATP pool. Thus, its elevated activity in ammonium-limited grown mycelia might be one reason for the lower ATP concentration in this phenotype (Figure 4A), although this does not explain the reduced NAD/NADH concentrations. Nevertheless, one has to be cautious when transferring this correlation between H^+^-ATPase activity and ATP pool to phosphate-limited cultures. Although this phenotype exhibits an even more decreased ATP level (Figure 4), this might also be a consequence of the limited availability of phosphate (Figure 7) and not due to an elevated ATP demand of the H^+^-ATPase.

As mentioned above evidence for changes in the plasma membrane of filamentous fungi in response to nutrient limitation is scarce (Meixner et al., 1985). Unpublished own results of a bioreactor batch culture of *P. ochrochloron* indicate that the plasma membrane responds strongly to medium composition. This is in line with studies from yeasts and bacteria, where there is a growing body of evidence that changes in environmental conditions have consequences for the composition and function of the plasma membrane (Markham et al., 1993; Monk et al., 1993; Krampe et al., 1998; Gaskova et al., 2002; Krampe and Boles, 2002; Turk et al., 2004, 2007; Rossell et al., 2005; Grossmann et al., 2006, 2007; Ferenci, 2007; Niittylae et al., 2007; Buziol et al., 2008; Kresnowati et al., 2008). Given the crucial function of the plasma membrane, these observations highlight the need to investigate this important organelle on the borderline between a living cell and its outside in more depth.

#### Interrelation of organic acid excretion and PM-M-R dynamics in response to different nutrient limitations

##### Glucose limitation

As a reference for the other phenotypes investigated, the PM-M-R dynamics of the glucose-limited phenotype showed some interesting aspects. Facing simultaneously a carbon and an energy limitation (Figure 7), glucose-limited grown mycelia of *P. ochrochloron* barely excreted any organic acids (Figure 3) due to the low glycolytic flux (Table 1; Gallmetzer and Burgstaller, 2001). A respiratory activity far from the maximum (Figure 5; Schinagl et al., 2016) also pointed at a restricted catabolic flux. This was combined with high nucleotide levels and a high EC (Figure 4), which seems to be rather typical for glucose-limited grown mycelia (e. g., Nasution et al., 2006b; Vrabl et al., 2009). One of the main ATP consumers in the cell, the plasma membrane H^+^-ATPase, was present but active at lower levels only (Figure 6), which might also partly explain the high level of ATP (Figure 4).

##### Ammonium limitation

Altogether, for ammonium-limited grown mycelia the following scenario emerged for the relationship between an increased organic acid excretion by this phenotype and the PM-M-R dynamics. Compared to glucose-limited mycelia these mycelia were in a physiological state of energy excess (Figure 7). These mycelia showed a higher consumption of oxygen and glucose, a lower concentration of ATP and a higher activity of plasma membrane H^+^-ATPase.

These findings are not unusual for fungi grown with nitrogen limitation. A lower concentration of ATP combined with an increased glycolytic flux during ammonium-limited growth has also been found after a relief from glucose limitation in the filamentous fungus *P. chrysogenum* (Nasution et al., 2006a) and in the yeast *Saccharomyces cerevisiae* (Koebmann et al., 2002; Koefoed et al., 2002; Özalp et al., 2010; Ytting et al., 2012) as well as during ammonium-limited steady state growth in *Klebsiella aerogenes* (Harrison and Maitra, 1969) and *S. cerevisiae* (Larsson et al., 1997). Özalp et al. (2010) and Ytting et al. (2012) both assumed that the reason for the lower ATP concentration was an increased ATP hydrolysis by plasma membrane ATPases.

This would coincide with our observation of an increased amount/activity of the plasma membrane H^+^-ATPase under these conditions. An increased organic acid excretion found with ammonium-limited growing mycelia could then be explained to satisfy the increased need of charge compensation for the increased proton excretion [or vice versa] by the plasma membrane H^+^-ATPase (Burgstaller, 2006).

In addition, the difference between glucose-limited grown mycelia and ammonium-limited grown mycelia concerning the plasma membrane ATPase seems rather to be a long term adaptation to nutrient supply, because in batch culture there was no change in plasma membrane H^+^-ATPase up to 5 h after glucose or ammonium were exhausted (Zimmer and Speckbacher, 2015). The same was reported for *S. cerevisiae* (Müller et al., 2015).

##### Phosphate limitation

From a wider perspective phosphate-limited grown mycelia are a special case since the phosphate deficiency limits ATP synthesis, which is reflected in the low levels of adenine nucleotides (Figure 4). Nevertheless, this phenotype exhibited high levels of organic acid excretion (Figure 3). Like for the other two phenotypes, the EC remained high, although it was a little bit lower, which supports our previous hypothesis that organic acid excretion is not triggered by the EC (Vrabl et al., 2009). Compared to glucose-limited grown mycelia phosphate-limited grown mycelia were furthermore characterized by: (i) a slightly lower rate of specific respiration (Table 1); (ii) an increased rate of glucose consumption (Table 1); and (iii) a distinct inhibition of AOX activity by SHAM after inhibition of the cytochrome pathway by azide (Figure 5). Also in *A. niger* there is evidence that under conditions of citric acid excretion a switch to alternative respiration takes place (Kubicek et al., 1980; Kirimura et al., 1987, 1996, 2000; Wallrath et al., 1991, 1992; Hattori et al., 2008). The elevated amount of excreted organic acids and the elevated activity of AOX in phosphate-limited grown mycelia might serve as a carbon and electron sink to keep a more or less unrestricted catabolic flux. The scenario found in *P. ochrochloron* resembles the one that was postulated for *E. coli* under increasingly phosphate-limited fed-batch conditions (Schuhmacher et al., 2014): first a decoupling of the ATP synthetase resulting in increased glucose uptake, then a shift to oxidases with less contribution to the proton motive force, and last excretion of acetate.

## Conclusions

This study emphasizes that many aspects in the physiology of filamentous fungi, especially primary metabolism, are currently only poorly understood (Andersen, 2014; Meyer et al., 2016). To explore organic acid excretion from a new point of view, we used a multi-level approach: For the first time organic acid excretion and its interrelation with the three important metabolic levels of plasma-membrane (PM), (nucleotide) metabolism (M) and respirometry (R) was studied simultaneously, quantitatively, and related to one another under various nutrient limitations (C-, N-, P-limitation). All target levels were affected in various degrees by each tested nutrient limitation. The effects ranged from distinct changes in physiological parameters like respiration rates or nucleotide content to differences in filtration behavior, sample extraction or aggregation behavior. In consequence, some experimental protocols could not be applied for all explored phenotypes without adaptation, or even failed completely. This means that analytical methods must be adapted at the strain level and sometimes even at the phenotype level.

As expected, organic acid excretion was considerably increased during ammonium- and phosphate-limited conditions. Compared to glucose-limited mycelium, the plasma membrane H^+^-ATPase activity was increased under ammonium limitation. While the studied energetic ratios such as EC or CRC appeared to be very stable over all nutrient limitations, we observed an inverse correlation of nucleotide contents and organic acid excretion. In *P. ochrochloron* the alternative oxidase was present in all tested experiments but appeared to be more important under phosphate-limited conditions. In a way, the results also highlighted the strong interdependency of the targeted cellular levels. Therefore, reducing the observed dynamics to only a few trigger factors or even a single factor is, to our opinion, hardly meaningful. Instead, through the strong linkage of the single target levels or metabolite pools with each other it is rather questionable to view them independently at all (Igamberdiev and Kleczkowski, 2009). In contrary, our data support the recent shift in perspective toward a more pleiotropic metabolic regulation (Igamberdiev and Kleczkowski, 2009; Mensonides et al., 2013; Chubukov et al., 2014). Obtaining reliable quantitative data to test this hypothesis is certainly a future challenge as the necessary methodological tools have yet to be developed.

## Author contributions

CS, PV, and WB conceived and designed the study. CS, DA, PV, and WB performed the bioreactor cultivations. DA and PV established and conducted the rapid sampling and metabolite extractions with assistance of AK, CS, and WB. AK established and performed the nucleotide analytics and analyzed these data together with DA, MG, and PV. CS established and performed the high resolution experiments with assistance of PV and WB and analyzed the data together with PV and CS. WB established and conducted the plasma membrane experiments with assistance of CS and PV. AP performed mass spectrometry of plasma membranes and analyzed the data together with WB. CS, PV, and WB drafted the manuscript with assistance of AK and AP. All authors critically revised the manuscript and approved its final version.

### Conflict of interest statement

The authors declare that the research was conducted in the absence of any commercial or financial relationships that could be construed as a potential conflict of interest.

## Abbreviations

AOX: Alternative Oxidase
ARC: Anabolic Reduction Charge
CCCP: carbonyl cyanide m- chlorophenylhydrazone
CRC: Catabolic Reduction Charge
DNP: 2,4-dinitrophenol
EC: Energy Charge
ETS: Electron Transport System
FCCP: carbonyl cyanide 4-(trifluoromethoxy) phenylhydrazone.

## References

[B1] Andersen M. R. (2014). Elucidation of primary metabolic pathways in *Aspergillus* species: orphaned research in characterizing orphan genes. Brief. Funct. Genomics 13, 451–455. 10.1093/bfgp/elu029 25114096 PMC4239788

[B2] Andersen M. R. Lehmann L. Nielsen J. (2009). Systemic analysis of the response of *Aspergillus niger* to ambient pH. Genome Biol. 10:R47. 10.1186/gb-2009-10-5-r47 19409083 PMC2718513

[B3] Avery S. V. (2005). Phenotypic diversity and fungal fitness. Mycologist 19, 74–80. 10.1017/S0269915X05002053

[B4] Bergmeyer H. U. (1974). Methoden der Enzymatischen Analyse, Band I, 3 Auflage. Weinheim: Verlag Chemie.

[B5] Braaksma M. Bijlsma S. Coulier L. Punt P. J. van der Werf M. J. (2011). Metabolomics as a tool for target identification in strain improvement: the influence of phenotype definition. Microbiology 157, 147–159. 10.1099/mic.0.041244-0 20847006

[B6] Bridge P. D. Hudson L. Kozakiewicz Z. Onions A. H. S. Paterson R. R. M. (1987). Investigation of variation in phenotype and DNA content between single-conidium isolates of single Penicillium Strains. J. Gen. Microbiol. 133, 995–1004. 10.1099/00221287-133-4-995 3655739

[B7] Brody S. (1972). Regulation of pyridine nucleotide levels and ratios in *Neurospora crassa*. J. Biol. Chem. 247, 6013–6017 4405599

[B8] Buziol S. Warth L. Magario I. Freund A. Siernann-Herzberg M. Reuss M. (2008). Dynamic response of the expression of Hxt1, Hxt5 and Hxt7 transport proteins in *Saccharomyces cerevisiae* to perturbations in the extracellular glucose concentration. J. Biotechnol. 134, 203–210. 10.1016/j.jbiotec.2008.02.002 18367282

[B9] Bull A. T. (2010). The renaissance of continuous culture in the post-genomics age. J. Ind. Microbiol. Biotechnol. 37, 993–1021. 10.1007/s10295-010-0816-4 20835748

[B10] Burgstaller W. (2006). Thermodynamic boundary conditions suggest that a passive transport step suffices for citrate excretion in Aspergillus and Penicillium. Microbiology 152, 887–893. 10.1099/mic.0.28454-0 16514167

[B11] Burgstaller W. Müller B. Schinner F. (1997). The *in vivo* effect of glucose and extracellular pH on the plasma membrane H+-ATPase of *Penicillium simplicissimum*. FEMS Microbiol. Lett. 147, 109–114. 10.1111/j.1574-6968.1997.tb10228.x

[B12] Chubukov V. Gerosa L. Kochanowski K. Sauer U. (2014). Coordination of microbial metabolism. Nat. Rev. Microbiol. 12, 327–340. 10.1038/nrmicro3238 24658329

[B13] da Luz J. A. Hans E. Zeng A.-P. (2014). Automated fast filtration and on-filter quenching improve the intracellular metabolite analysis of microorganisms. Eng. Life Sci. 14, 135–142. 10.1002/elsc.201300099

[B14] Dawson R. M. C. Elliott D. C. Elliott W. H. Jones K. M. (1986). Data for Biochemical Research, 3^*rd*^ Edn. Oxford: Oxford Science Publications, Clarendon Press.

[B15] Dengg H. (2014). Vanadate Sensitive ATPase Activity in the Plasma Membrane of Penicillium ochrochloron Grown in Glucose Limited Chemostat. Master Thesis, University of Innsbruck.

[B16] Doblander C. Lackner R. (1996). Metabolism and detoxification of nitrite by trout hepatocytes. Biochim. Biophys. Acta 1289, 270–274. 8600984 10.1016/0304-4165(95)00166-2

[B17] Dorfer V. Pichler P. Stranzl T. Stadlmann J. Taus T. Winkler S.. (2014). MS Amanda, a universal identification algorithm optimized for high accuracy tandem mass spectra. J. Proteome Res. 13, 3679–3684. 10.1021/pr500202e 24909410 PMC4119474

[B18] Dyballa N. Metzger S. (2009). Fast and sensitive colloidal coomassie G-250 staining for proteins in polyacrylamide gels. J. Vis. Exp. 30:e1431 10.3791/1431 PMC3149902 19684561

[B19] Egli T. (1991). On multiple-nutrient-limited growth of microorganisms, with special reference to dual limitation by carbon and nitrogen substrates. A van Leeuwenhoek 60, 225–234. 10.1007/BF00430367 1687236

[B20] Egli T. (2015). Microbial growth and physiology: a call for better craftsmanship. Front. Microbiol. 6:287. 10.3389/fmicb.2015.00287 25926822 PMC4396425

[B21] Eng J. K. McCormack A. L. Yates J. R. (1994). An approach to correlate tandem mass spectral data of peptides with amino acid sequences in a protein database. J. Am. Soc. Mass Spectrometry 5, 976–989. 10.1016/1044-0305(94)80016-2 24226387

[B22] Ferenci T. (2007). Bacterial physiology, regulation and mutational adaptation in a chemostat environment, in Advances in Microbial Physiology, Vol. 53, ed Poole R. K. (San Diego, CA: Academic Press), 169–315. 10.1016/S0065-2911(07)53003-1 17707145

[B23] Foster J. W. (1949). Chemical Activities of Fungi. New York, NY: Academic Press.

[B24] Franz A. Burgstaller W. Schinner F. (1991). Leaching with *Penicillium simplicissimum*: influence of metals and buffers on proton extrusion and citric acid production. Appl. Environ. Microbiol. 57, 769–774. 16348442 10.1128/aem.57.3.769-774.1991 PMC182793

[B25] Führer L. Kubicek C. P. Röhr M. (1980). Pyridine nucleotide levels and ratios in *Aspergillus niger*. Can. J. Microbiol. 26, 405–408. 10.1139/m80-067 7407716

[B26] Gallmetzer M. Burgstaller W. (2001). Citrate efflux in glucose-limited and glucose-sufficient chemostat culture of *Penicillium simplicissium*. A van Leeuwenhoek 79, 81–87. 10.1023/A:1010295924549 11392488

[B27] Gallmetzer M. Burgstaller W. (2002). Efflux of organic acids in *Penicillium simplicissimum* is an energy-spilling process, adjusting the catabolic carbon flow to the nutrient supply and the activity of catabolic pathways. Microbiology 148, 1143–1149. 10.1099/00221287-148-4-1143 11932458

[B28] Ganzera M. Vrabl P. Wörle E. Burgstaller W. Stuppner H. (2006). Determination of adenine and pyridine nucleotides in glucose-limited chemostat cultures of *Penicillium simplicissimum* by one-step ethanol extraction and ion-pairing liquid chromatography. Anal. Biochem. 359, 132–140. 10.1016/j.ab.2006.09.012 17054897

[B29] Garcia J. Torres N. (2011). Mathematical modelling and assessment of the pH homeostasis mechanisms in *Aspergillus niger* while in citric acid producing conditions. J. Theor. Biol. 282, 23–35. 10.1016/j.jtbi.2011.04.028 21549718

[B30] Gaskova D. Cadek R. Chaloupka R. Vacata V. Gebel J. Sigler K. (2002). Monitoring the kinetics and performance of yeast Membrane ABC transporters by Dis-C-3(3) fluorescence. Int. J. Biochem. Cell Biol. 34, 931–937. 10.1016/S1357-2725(02)00013-4 12007631

[B31] Gasser E. (2014). Effects of Nutrient Limitations on the Plasma Membrane H^+^*-ATPase of Penicillium ochrochloron* Master Thesis, University of Innsbruck.

[B32] Gonzalez B. Francois J. Renaud M. (1997). A rapid and reliable method for metabolite extraction in yeast using boiling buffered ethanol. Yeast 13, 1347–1355. 10.1002/(SICI)1097-0061(199711)13:14<1347::AID-YEA176>3.0.CO2-O 9392079

[B33] Grossmann G. Opekarova M. Malinsky J. Weig-Meckl I. Tanner W. (2007). Membrane potential governs lateral segregation of plasma membrane proteins and lipids in yeast. EMBO J. 26, 1–8. 10.1038/sj.emboj.7601466 17170709 PMC1782361

[B34] Grossmann G. Opekarova M. Novakova L. Stolz J. Tanner W. (2006). Lipid raft-based membrane compartmentation of a plant transport protein expressed in *Saccharomyces cerevisiae*. Eukaryot. Cell 5, 945–953. 10.1128/EC.00206-05 16757742 PMC1489273

[B35] Hajjaj H. Blanc P. Goma G. Francois J. (1998). Sampling techniques and comparative extraction procedures for quantitative determination of intra- and extracellular metabolites in filamentous fungi. FEMS Microbiol. Lett. 164, 195–200. 10.1111/j.1574-6968.1998.tb13085.x

[B36] Harrison D. E. Maitra P. K. (1969). Control of respiration and metabolism in growig Klebsiella aerogenes - role of adenine nucleotides. Biochem. J. 112, 647–656. 10.1042/bj1120647 4309671 PMC1187768

[B37] Hattori T. Honda Y. Kino K. Kirimura K. (2008). Expression of alternative oxidase gene (aox1) at the stage of single-cell conidium in citric acid-producing *Aspergillus niger*. J. Biosci. Bioeng. 105, 55–57. 10.1263/jbb.105.55 18295720

[B38] Hewitt S. K. Foster D. S. Dyer P. S. Avery S. V. (2016). Phenotypic heterogeneity in fungi: importance and methodology. Fungal Biol. Rev. 30, 176–184. 10.1016/j.fbr.2016.09.002

[B39] Igamberdiev A. U. Kleczkowski L. A. (2009). Metabolic systems maintain stable non-equilibrium via thermodynamic buffering. Bioessays 31, 1091–1099. 10.1002/bies.200900057 19708023

[B40] Jernejc K. Legisa M. (2001). Activation of plasma membrane H^+^-ATPase by ammonium ions in *Aspergillus niger*. Appl. Microbiol. Biotechnol. 57, 368–373. 10.1007/s002530100697 11759687

[B41] Kim K. S. Yoo Y. D. Kim M. H. (1995). Control of intracellular ammonium level using specific oxygen uptake rate for overproduction of citric acid by *Aspergillus niger*. J. Ferment. Bioeng. 79, 555–559. 10.1016/0922-338X(95)94747-F

[B42] Kirimura K. Kirowatari Y. Usami S. (1987). Alterations of respiratory systems in *Aspergillus niger* under the conditions of citric acid fermentation. Agric. Biol. Chem. 51, 1299–1303. 10.1271/bbb1961.51.1299

[B43] Kirimura K. Matsui T. Sugano S. Usami S. (1996). Enhancement and repression of cyanide-insensitive respiration in *Aspergillus niger*. FEMS Microbiol. Lett. 141, 251–254. 10.1111/j.1574-6968.1996.tb08393.x 8768530

[B44] Kirimura K. Yoda M. Shimizu M. Sugano S. Mizuno M. Kino K.. (2000). Contribution of a cyanide-insensitive respiratory pathway, catalyzed by the alternative oxidase, to citric acid production in *Aspergillus niger*. Biosci. Biotechnol. Biochem. 64, 2034–2039. 10.1271/bbb.64.2034 11129572

[B45] Knuf C. Nookaew I. Brown S. H. McCulloch M. Berry A. Nielsen J. (2013). Investigation of malic acid production in *Aspergillus oryzae* under nitrogen starvation conditions. Appl. Environ. Microbiol. 79, 6050–6058. 10.1128/AEM.01445-13 23892740 PMC3811345

[B46] Koebmann B. J. Westerhoff H. V. Snoep J. L. Solem C. Pedersen M. B. Nilsson D.. (2002). The extent to which ATP demand controls the glycolytic flux depends strongly on the organism and conditions for growth. Mol. Biol. Rep. 29, 41–45. 10.1023/A:1020398117281 12241072

[B47] Koefoed S. Otten M. Koebmann B. Bruggeman F. Bakker B. Snoep J.. (2002). A turbo engine with automatic transmission? How to marry chemicomotion to the subtleties and robustness of life. Biochim. Biophys. Acta 1555, 75–82. 10.1016/S0005-2728(02)00258-X 12206895

[B48] Krampe S. Boles E. (2002). Starvation-induced degradation of yeast hexose transporter Hxt7p is dependent on endocytosis, autophagy and the terminal sequences of the permease. FEBS Lett. 513, 193–196. 10.1016/S0014-5793(02)02297-4 11904149

[B49] Krampe S. Stamm O. Hollenberg C. P. Boles E. (1998). Catabolite inactivation of the high-affinity hexose transporters Hxt6 and Hxt7 of *Saccharomyces cerevisiae* occurs in the vacuole after internalization by endocytosis. FEBS Lett. 441, 343–347. 10.1016/S0014-5793(98)01583-X 9891967

[B50] Kresnowati M. T. A. P. Van Winden W. A. Van Gulik W. M. Heijnen J. J. (2008). Dynamic *in vivo* metabolome response of *Saccharomyces cerevisiae* to a stepwise perturbation of the ATP requirement for benzoate export. Biotechnol. Bioeng. 99, 421–441. 10.1002/bit.21557 17614335

[B51] Krüger A. (2013). Analysis of Metabolites in Plants and Filamentous Fungi, on the Examples of Oroxylum indicum and Penicillium ochrochloron. PhD Thesis, University of Innsbruck.

[B52] Krull R. Cordes C. Horn H. Kampen I. Kwade A. Neu T. R.. (2010). Morphology of filamentous fungi: linking cellular biology to process engineering using *Aspergillus niger*. Adv. Biochem. Eng. Biotechnol. 121, 1–21. 10.1007/10_2009_60 20490972

[B53] Kubicek C. P. Zehentgruber O. El-Kalak H. M. R. (1980). Regulation of citric acid production by oxygen: effect of dissolved oxygen tension on adenylate levels and respiration in *Aspergillus niger*. Eur. J. Appl. Microbiol. Biotechnol. 9, 101–115. 10.1007/BF00503505

[B54] Lambowitz A. M. Slayman C. W. (1971). Cyanide-resistant respiration in *Neurospora crassa*. J. Bacteriol. 108, 1087–1096. 4333318 10.1128/jb.108.3.1087-1096.1971 PMC247191

[B55] Lange H. C. Eman M. Van Zuijlen G. Visser D. Van Dam J. C. Frank J.. (2001). Improved rapid sampling for *in vivo* kinetics of intracellular metabolites in *Saccharomyces cerevisiae*. Biotechnol. Bioeng. 75, 406–415. 10.1002/bit.10048 11668440

[B56] Lanzetta P. A. Alvarez J. L. Reinach P. S. Candia O. A. (1979). An improved assay for nanomole amounts of inorganic phosphate. Anal Biochem 100, 95–97. 161695 10.1016/0003-2697(79)90115-5

[B57] Larsson C. Nilsson A. Blomberg A. Gustafsson L. (1997). Glycolytic flux is conditionally correlated with ATP concentration in *Saccharomyces cerevisiae*: a chemostat study under carbon- or nitrogen-limiting conditions. J. Bacteriol. 179, 7243–7250. 10.1128/jb.179.23.7243-7250.1997 9393686 PMC179672

[B58] Larsson C. Widell S. (2000). Isolation of plant plasma membranes and production of inside-out vesicles, in Methods in Biotechnology, Vol. 11, Aqueous Two-Phase Systems: Methods and Protocols, ed Hatti-Kaul R. (Totowa, NJ: Humana Press Inc.), 159–166.

[B59] Markham P. Robson G. D. Bainbridge B. W. Trinci A. P. J. (1993). Choline: its role in the growth of filamentous fungi and the regulation of mycelial morphology. FEMS Microbiol. Rev. 104, 287–300. 10.1111/j.1574-6968.1993.tb05872.x 8318261

[B60] Mason H. R. S. Righelato R. C. (1976). Energetics of fungal growth: the effect of growth-limiting substrate on respiration of *Penicillium chrysogenum*. J. Appl. Chem. Biotech. 26, 145–152. 10.1002/jctb.5020260308

[B61] McGetrick A. M. T. Bull A. T. (1979). Phenotypic changes in the chemistry of *Aspergillus nidulans*: influence of culture conditions on mycelial composition. Arch. Microbiol. 123, 151–156. 10.1007/BF00446814 44181

[B62] Meixner O. Mischak H. Kubicek C. P. Röhr M. (1985). Effect of manganese deficiency on plasma-membrane lipid composition and glucose uptake in *Aspergillus niger*. FEMS Microbiol. Lett. 26, 271–274. 10.1111/j.1574-6968.1985.tb01609.x

[B63] Mensonides F. I. C. Bakker B. M. Cremazy F. Messiha H. L. Mendes P. Boogerd F. C.. (2013). A new regulatory principle for *in vivo* biochemistry: Pleiotropic low affinity regulation by the adenine nucleotides - Illustrated for the glycolytic enzymes of *Saccharomyces cerevisiae*. FEBS Lett. 587, 2860–2867. 10.1016/j.febslet.2013.07.013 23856461

[B64] Meyer V. Andersen M. R. Brakhage A. A. Braus G. H. Caddick M. X. Cairns T. C.. (2016). Current challenges of research on filamentous fungi in relation to human welfare and a sustainable bio-economy: a white paper. Fungal Biol. Biotechnol. 3, 6. 10.1186/s40694-016-0024-8 28955465 PMC5611618

[B65] Monk B. C. Niimi M. Shepherd M. G. (1993). The *Candida albicans* plasma membrane and H^+^-ATPase during yeast growth and germ tube formation. J. Bacteriol. 175, 5566–5574. 10.1128/jb.175.17.5566-5574.1993 8366041 PMC206613

[B66] Mosley M. J. Pitt D. Barnes J. C. (1989). Adenine and pyridine nucleotide levels during calcium-induced conidiation in *Penicillium notatum*. Antonie Van Leeuwenhoek 56:191. 10.1007/BF00399982 2802576

[B67] Müller B. Zanella A. Burgstaller W. (2001). A method to obtain right-side-out and sealed plasma membrane vesicles from the filamentous fungus *Penicillium simplicissimum*. J. Basic Microbiol. 41, 281–288. 10.1002/1521-4028(200110)41:5<281::AID-JOBM281>3.0.CO2-N 11688214

[B68] Müller M. Schmidt O. Angelova M. Faserl K. Weys S. Kremser L.. (2015). The coordinated action of the MVB pathway and autophagy ensures cell survival during starvation. Elife 4:e07736. 10.7554/eLife.07736 25902403 PMC4424281

[B69] Nasution U. van Gulik W. M. Kleijn R. J. van Winden W. A. Proell A. Heijnen J. J. (2006b). Measurement of intracellular metabolites of primary metabolism and adenine nucleotides in chemostat cultivated *Penicillium chrysogenum*. Biotechnol. Bioeng. 94, 159–166. 10.1002/bit.20842 16508996

[B70] Nasution U. van Gulik W. M. Proell A. van Winden W. A. Heijnen J. J. (2006a). Generating short-term kinetic responses of primary metabolism of *Penicillium chrysogenum* through glucose perturbation in the bioscope mini reactor. Metab. Eng. 8, 395–405. 10.1016/j.ymben.2006.04.002 16807032

[B71] Neijssel O. M. Texeira de Mattos M. J. Tempest D. W. (1993). Overproduction of metabolites, in Biotechnology, Vol. I, *Biological Fundamentals*, eds Rehm H.-J. Reed G. (Weinheim: VCH), 163–187.

[B72] Niittylae T. Fuglsang A. T. Palmgren M. G. Frommer W. B. Schulze W. X. (2007). Temporal analysis of sucrose-induced phosphorylation changes in plasma membrane proteins of Arabidopsis. Mol. Cell. Proteomics 6, 1711–1726. 10.1074/mcp.M700164-MCP200 17586839

[B73] Özalp V. C. Pedersen D. R. Nielsen L. J. Olsen L. F. (2010). Time-resolved measurements of intracellular ATP in the yeast *Saccharomyces cerevisiae* using a new type of nanobiosensor. J. Biol. Chem. 285, 37579–37588. 10.1074/jbc.M110.155119 20880841 PMC2988363

[B74] Pitt D. E. Bull A. T. (1982). The adenine nucleotide composition of growing and stressed cultures of *Trichoderma aureoviride*. Exp. Mycol. 6, 41–5110. 10.1016/0147-5975(82)90062-7

[B75] Plassard C. Fransson P. (2009). Regulation of low-molecular weight organic acid production in fungi. Fungal Biol. Rev. 23, 30–39. 10.1016/j.fbr.2009.08.002

[B76] Rayner A. D. M. (1996). Interconnectedness and individualism in fungal mycelia, in A Century of Mycology, ed Sutton B. (Cambridge: Cambridge University Press), 193–232.

[B77] Read S. M. Northcote D. H. (1981). Minimization of variation in the response to different proteins of the Coomassie Blue G dye-binding assay for protein. Anal Biochem. 116, 53–64. 7304986 10.1016/0003-2697(81)90321-3

[B78] Roberts S. K. Milnes J. Caddick M. (2011). Characterisation of AnBEST1, a functional anion channel in the plasma membrane of the filamentous fungus *Aspergillus nidulans*. Fungal Gen. Biol. 48, 928–938. 10.1016/j.fgb.2011.05.004 21596151

[B79] Rosenfeld E. Beauvoit B. (2003). Role of the non-respiratory pathways in the utilization of molecular oxygen by *Saccharomyces cerevisiae*. Yeast 20, 1115–1144. 10.1002/yea.1026 14558145

[B80] Rossell S. Van Der Weijden C. C. Kruckeberg A. L. Bakker B. M. Westerhoff H. V. (2005). Hierarchical and metabolic regulation of glucose influx in starved *Saccharomyces cerevisiae*. FEMS Yeast Res. 5, 611–619. 10.1016/j.femsyr.2004.11.003 15780660

[B81] Ruijter G. J. G. Kubicek C. P. Visser J. (2002). Production of organic acids by fungi, in Industrial Applications, ed Osiewacz H. D. (Berlin: Springer), 213–230.

[B82] Ruijter G. J. G. Visser J. (1996). Determination of intermediary metabolites in *Aspergillus niger*. J. Microbiol. Methods 25, 295–302.

[B83] Russell J. B. (2007). The energy spilling reactions of bacteria and other organisms. J. Mol. Microbiol. Biotechnol. 13, 1–11. 10.1016/0167-7012(95)00104-2 17693707

[B84] Sanders D. (1988). Fungi, in Solute Transport in Plant Cells and Tissues, eds Baker D. A. Hall J. L. (Harlow: Longman Scientific and Technical), 106–165.

[B85] Sauer M. Porro D. Mattanovich D. Branduardi P. (2008). Microbial production of organic acids: expanding the markets. Trends Biotechnol. 26, 100–108. 10.1016/j.tibtech.2007.11.006 18191255

[B86] Schinagl C. W. Vrabl P. Burgstaller W. (2016). Adapting high-resolution respirometry to glucose-limited steady state mycelium of the filamentous fungus *Penicillium ochrochloron*: method development and standardisation. PLoS ONE 11:e0146878. 10.1371/journal.pone.0146878 26771937 PMC4714917

[B87] Schluesener D. Fischer F. Kruip J. Rogner M. Poetsch A. (2005). Mapping the membrane proteome of *Corynebacterium glutamicum*. Proteomics 5, 1317–1330. 10.1002/pmic.200400993 15717325

[B88] Schuhmacher T. Löffler M. Hurler T. Takors R. (2014). Phosphate limited fed-batch processes: impact on carbon usage and energy metabolism in *Escherichia coli*. J. Biotechnol. 190, 96–104. 10.1016/j.jbiotec.2014.04.025 24833421

[B89] Siedow J. N. Umbach A. L. (2000). The mitochondrial cyanide-resistant oxidase: structural conservation amid regulatory diversity. Biochim. Biophys. Acta 1459, 432–439. 10.1016/S0005-2728(00)00181-X 11004460

[B90] Silva J. C. Gorenstein M. V. Li G. Z. Vissers J. P. Geromanos S. J. (2006). Absolute quantification of proteins by LCMSE: a virtue of parallel MS acquisition. Mol. Cell. Proteomics 5, 144–156. 10.1074/mcp.M500230-MCP200 16219938

[B91] Slayman C. L. (1973). Adenine nucleotide levels in Neurospora, as influenced by conditions of growth and by metabolic inhibitors. J. Bacteriol. 114, 752–766. 4267534 10.1128/jb.114.2.752-766.1973 PMC251836

[B92] Slayman C. L. Kaminski P. Stetson D. (1990). Structure and function of fungal plasma-membrane ATPases, in Biochemistry of Cell Walls and Membranes in Fungi, eds Kuhn P. J. Trinci A. P. J. Jung M. J. Goosey M. W. Copping L. G. (Berlin: Springer Verlag), 299–316.

[B93] Slepecky R. A. Starmer W. T. (2009). Phenotypic plasticity in fungi: a review with observations on *Aureobasidium pullulans*. Mycologia 101, 823–832. 10.3852/08-197 19927747

[B94] Sluse F. E. Jarmuszkiewicz W. (1998). Alternative oxidase in the branched mitochondrial respiratory network: an overview on structure, function, regulation, and role. Braz. J. Med. Biol. Res. 31, 733–747. 10.1590/S0100-879X1998000600003 9698817

[B95] Steiger M. G. Blumhoff M. L. Mattanovich D. Sauer M. (2013). Biochemistry of microbial itaconic acid production. Front. Microbiol. 4:23. 10.3389/fmicb.2013.00023 23420787 PMC3572532

[B96] Stephanopoulos G. N. Aristidou A. A. Nielsen J. (1998). CHAPTER 5 - Regulation of Metabolic Pathways, In Metabolic Engineering. San Diego, CA: Academic Press.

[B97] Tempest D. W. Neijssel O. M. (1992). Physiological and energetic aspects of bacterial metabolite overproduction. FEMS Microbiol. Lett. 100, 169–176. 10.1111/j.1574-6968.1992.tb05699.x 1478453

[B98] Turk M. Mejanelle L. Sentjurc M. Grimalt J. O. Gunde-Cimerman N. Plemenitas A. (2004). Salt-induced changes in lipid composition and membrane fluidity of halophilic yeast-like melanized fungi. Extremophiles 8, 53–61. 10.1007/s00792-003-0360-5 15064990

[B99] Turk M. Montiel V. Zigon D. Plemenitas A. Ramos J. (2007). Plasma membrane composition of *Debaryomyces hansenii* adapts to changes in pH and external salinity. Microbiology 153, 3586–3592. 10.1099/mic.0.2007/009563-0 17906155

[B100] Vera M. Krok B. Bellenberg S. Sand W. Poetsch A. (2013). Shotgun proteomics study of early biofilm formation process of *Acidithiobacillus ferrooxidans* ATCC 23270 on pyrite. Proteomics 13, 1133–1144. 10.1002/pmic.201200386 23319327

[B101] Vrabl P. Artmann D. J. Schinagl C. W. Burgstaller W. (2016). Rapid sample processing for intracellular metabolite studies in *Penicillium ochrochloron* CBS 123.824: the FiltRes-device combines cold filtration of methanol quenched biomass with resuspension in extraction solution. Springerplus 5, 966. 10.1186/s40064-016-2649-8 27429876 PMC4932030

[B102] Vrabl P. Fuchs V. Pichler B. Schinagl C. W. Burgstaller W. (2012). Organic acid excretion in *Penicillium ochrochloron* increases with ambient pH. Front. Microbiol. 3:121. 10.3389/fmicb.2012.00121 22493592 PMC3318189

[B103] Vrabl P. Mutschlechner W. Burgstaller W. (2008). Characteristics of glucose uptake by glucose- and NH4-limited grown *Penicillium ochrochloron* at low, medium and high glucose concentration. Fungal Gen. Biol. 45, 1380–1392. 10.1016/j.fgb.2008.07.017 18722543

[B104] Vrabl P. Mutschlechner W. Burgstaller W. (2009). Dynamics of energy charge and adenine nucleotides during uncoupling of catabolism and anabolism in *Penicillium ochrochloron*. Mycol. Res. 113, 1422–1432. 10.1016/j.mycres.2009.09.011 19818403

[B105] Walder K. (2011). Optimierung der Gewinnung einer Mikrosomalen Fraktion aus Penicillium ochrochloron. Master Thesis, University of Innsbruck.

[B106] Wallrath J. Schmidt M. Weiss H. (1991). Concomitant loss of respiratory chain NADH:ubiquinone reductase (complex I) and citric acid accumulation in *Aspergillus niger*. Appl. Microbiol. Biotechnol. 36, 76–81. 10.1007/BF00164702

[B107] Wallrath J. Schmidt M. Weiss H. (1992). Correlation between manganese-deficiency, loss of respiratory chain complex I activity and citric acid production in *Aspergillus niger*. Arch. Microbiol. 158, 435–438. 10.1007/BF00276305

[B108] Witteveen C. B. F. Weber F. Busink R. Visser J. (1994). Isolation and characterization of two xylitol dehydrogenases from *Aspergillus niger*. Microbiology 140, 1679–1685. 10.1099/13500872-140-7-1679

[B109] Wösten H. A. B. van Veluw G. J. de Bekker C. Krijgsheld P. (2013). Heterogeneity in the mycelium: implications for the use of fungi as cell factories. Biotechnol. Lett. 35, 1155–1164. 10.1007/s10529-013-1210-x 23592308

[B110] Ytting C. K. Fuglsang A. T. Hiltunen J. K. Kastaniotis A. J. Özalp V. C. Nielsen L. J. Olsen L. F. (2012). Measurements of intracellular ATP provide new insight into the regulation of glycolysis in the yeast *Saccharomyces cerevisiae*. Integr Biol-Uk 4, 99–107. 10.1039/C1IB00108F 22134619

[B111] Zakhartsev M. Vielhauer O. Horn T. Yang X. Reuss M. (2014). Fast sampling for quantitative microbial metabolomics: new aspects on cold methanol quenching: metabolite co-precipitation. Metabolomics 11, 286–301. 10.1007/s11306-014-0700-8

[B112] Zimmer J. Speckbacher V. (2015). Charakterisierung von Penicillium ochrochloron in Glukose-, Ammonium, Sauerstoff- und Phosphatlimitierter Batch Kultur – Nährstoffumsatz, Wachstum, Säureausscheidung, Morphologie, Plasmamembranaufreinigung und Ionentransport. Master Thesis, University of Innsbruck.

