# Supplementary material for: The Dynamics of Plasma Membrane, Metabolism and Respiration (PM-M-R) in Penicillium ochrochloron CBS 123824 in Response to Different Nutrient Limitations—A Multi-level Approach to Study Organic Acid Excretion in Filamentous Fungi
Source: Front Microbiol. 2017 Dec 12;8:2475. doi: 10.3389/fmicb.2017.02475 (PMC5732977; doi:10.3389/fmicb.2017.02475)
Supplement: Supplementary file 1 [file DataSheet1.DOCX]

***Supplementary Material***

**The Dynamics of Plasma Membrane, Metabolism and Respiration (PM-M-R) in *Penicillium ochrochloron* CBS 123.824 in Response to Different Nutrient Limitations – a Multi-level Approach to Study Organic Acid Excretion in Filamentous Fungi**

Authors: Pamela Vrabl^1^**^*^**, Christoph W. Schinagl^1^**^*^**, Desirée J. Artmann^1^, Anja Krüger^2^, Markus Ganzera^2^, Ansgar Pötsch^3^ and Wolfgang Burgstaller^1**^

Affiliation: ^1^ University of Innsbruck, Institute of Microbiology, Technikerstrasse 25,
6020 Innsbruck, Austria

^2^ University of Innsbruck, Institute of Pharmacy/Pharmacognosy, Innrain 80-82,
6020 Innsbruck, Austria

^3^ Ruhr University Bochum, Plant Biochemistry, Universitätsstrasse 150,
44780 Bochum, Germany

***** These authors contributed equally to the work.

** Corresponding author: University of Innsbruck, Institute of Microbiology, Technikerstrasse 25, 6020 Innsbruck, Austria; Tel +43-512-507-51234; Fax +43-512-507-2929; [wolfgang.burgstaller@uibk.ac.at](mailto:wolfgang.burgstaller@uibk.ac.at)

**1. Section Growth Media and Chemicals**

Supplementary Table 1.1 Chemostat medium for glucose limitation

| Reagens | MW | mM |
| --- | --- | --- |
|  |  |  |
| Glucose 🞄 1 H_2_O | 198,17 | 20,00 |
| (NH_4_)_2_SO_4_ | 132,14 | 12,50 |
| KH_2_PO_4_ | 136,09 | 5,80 |
| MgSO_4_ 🞄 7 H_2_O | 246,48 | 1,60 |

Supplementary Table 1.2 Chemostat medium for phosphate limitation

| Reagens | MW | mM |
| --- | --- | --- |
| Glucose 🞄 1 H_2_O | 198,17 | 200,00 |
| (NH4)_2_SO_4_ | 132,14 | 6,00 |
| KH_2_PO_4_ | 136,09 | 0,11 |
| MgSO_4_ 7 H_2_O | 246,48 | 1,60 |
| KCl | 74,56 | 5,69 |

Supplementary Table 1.3 Chemostat medium for ammonia limitation

| Reagens | MW | mM |
| --- | --- | --- |
| Glucose 🞄 1 H_2_O | 198,17 | 200,00 |
| (NH_4_)_2_SO_4_ | 132,14 | 1,00 |
| KH_2_PO_4_ | 136,09 | 5,80 |
| MgSO_4_ 🞄 7 H_2_O | 246,48 | 1,60 |

Supplementary Table 1 4 Chemostat medium for nitrate limitation

| Reagens | MW | mM |
| --- | --- | --- |
| Glucose 🞄 1 H_2_O | 198,17 | 200,00 |
| KNO_3_ | 101,11 | 2,00 |
| KH_2_PO_4_ | 136,09 | 3,80 |
| Na_2_HPO_4_ 🞄 2 H_2_O | 177,99 | 2,00 |
| MgSO_4_ 7 H_2_O | 246,48 | 1,60 |

The **trace element solution** consisted of (mM): Fe (II)SO_4_ 7H_2_O 3.59, Mn(II) SO_4_ 1H_2_O 2.72, ZnCl_2_ 2.935, Cu (II) SO_4_ 5H_2_O 0.40 and CaCl_2_ 2H_2_O 4.08.

**Buffers**

CAPS, 3-(Cyclohexylamino)-1-propanesulfonic acid;

CHES, 2-(Cyclohexylamino)ethanesulfonic acid;

CTAB, Cetyltrimethylammoniumbromide;

EPPS, 3-[4-(2-Hydroxyethyl)piperazin-1-yl]propane-1-sulfonic acid, Sigma;

HEPES, 2-[4-(2-hydroxyethyl)piperazin-1-yl]ethanesulfonic acid;

TAPS, 3-[[1,3-dihydroxy-2-(hydroxymethyl)propan-2-yl]amino]propane-1-sulfonic acid;

**2. Section Plasma Membrane**

**Plasma membrane purification**

Supplementary Table 2.1 Disruption of hyphae and resuspension of membranes

| Homogenization medium (HM) | | Resuspension medium 1  (RM 1) | | Resuspension medium 2  (RM 2) | |
| --- | --- | --- | --- | --- | --- |
|  |  | for membranes to be loaded onto | | for membranes to be assayed | |
|  |  | the two phase system | | for vanadate sensitive ATPase activity | |
|  | Final conc. |  | Final conc. |  | Final conc. |
| HEPES pH 7.5  (10 M NaOH) | 50 mM | K-Phosphate buffer K_2_HPO_4_/KH_2_PO_4_ pH 7.8 at 4 °C | 200 mM | MES pH 6.0  (10 M NaOH) | 50 mM |
| Sucrose | 330 mM | Sucrose | 330 mM | Sucrose | 330 mM |
| Na-EDTA (Titriplex III) | 1 mM |  |  |  |  |
| Added shortly prior to use: |  | Added shortly prior to use: |  | Added shortly prior to use: |  |
| Bovine Serum Albumin (BSA), fatty acid free | 0.2 % (w/v) | Na-EDTA  (Titriplex III) | 0.1 mM | Na-EDTA  Titriplex III) | 0.1 mM |
| Dithiothreitol (DTT) | 3 mM | Dithiothreitol (DTT) | 1 mM | Dithiothreitol (DTT) | 1 mM |
| Ascorbic acid | 5 mM |  |  |  |  |
| Protease inhibitor cocktail Complete (Roche) | 1 tablet /100 mL |  |  |  |  |
| PMSF | 2 mM |  |  |  |  |

Supplementary  Tab. 2.2 Aqueous polymer two phase system

|  | Stock solutions |  |
| --- | --- | --- |
| Dextran T 500 | 20 % (w/w!) | Preparation Larsson and Widell 2000. Actual concentration determined with a polarimeter. |
| Polyethylene glycol | 40 % (w/w!) |  |
| KCl | 2 M |  |
| DTT/EDTA | 0.5 M/0.05 M | added shortly prior to use to give final conc. of 1 mM/0.1 mM (0.072 mL for a 36 g system) |
| K-Phosphate buffer | 0.2 M | see RM 1 |

**Mixing of the separation two phase system:**

in a 50 mL polycarbonate centrifuge tube with screw cap on a balance. Phase partitioning is temperature dependent. Solutions should be equilibrated at 4 °C, held on ice and all procedures should be performed at 4 °C.

Supplementary  Table 2.3 Separation mixture

|  | gram | final conc. | calc. on the basis of |
| --- | --- | --- | --- |
| Sucrose | 3.05 g | 330 mM | 27 g |
| Dextran | 11.295 g | 6.1 % (w/w) | 36 g, for a dextran stock solution of 19.443 % (w/w) |
| Polyethyleneglycol | 5.49 g | 6.1 % (w/w) | 36 g |
| Phosphate buffer | 0.675 mL | 5 mM | 27 g |
| KCl | 0.036 mL | 2 mM | 36 g |
| Deionized water | up to 27 g |  |  |

**Mixing of the washing two phase system in a separating funnel**

Supplementary Tab. 2.3  Washing mixture

|  | gram | final conc. | calc. on the basis of |
| --- | --- | --- | --- |
| Sucrose | 16.95 g | 330 mM | 150 g |
| Dextran | 47.06 g | 6.1 % (w/w) | 150 g |
| Polyethyleneglycol | 22.876 g | 6.1 % (w/w) | 150 g |
| Phosphate buffer | 3.75 mL | 5 mM | 150 g |
| KCl | 0.15 mL | 2 mM | 150 g |
| Deionized water | up to 150 g |  |  |

**Loading of the two phase system on a balance, mixing, and separation of phases**

- Add DTT/EDTA to the 27 g system.
- Add microsomal fraction re-suspended in RM 1 (72 mg of protein at maximum for a 36 g system).
- Carefully stir the microsomal fraction into the upper phase.
- Fill with RM 1 to 36 g.
- Incubate on ice for 5 minutes.
- Mix thoroughly by 40 inversions of the tube by hand.
- Centrifuge at 1500 g for 5 minutes in a swinging bucket rotor for phase separation.

**Biomass storage**


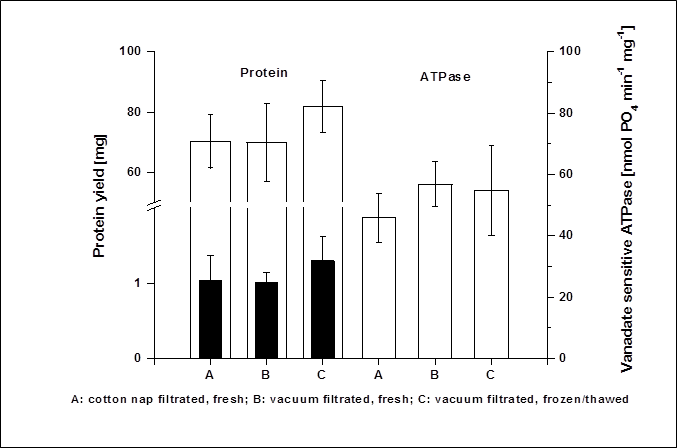


Supplementary Figure 2.1 Biomass storage. Effect of storage at -20 °C on the vanadate sensitive ATPase activity in a microsomal fraction *of Penicillium ochrochloron*. Protein / unfilled bars: protein yield in the crude extract; Protein / black bars: protein yield in the microsomal fraction.

**Cell Disruption**

Supplementary Table 2.4 Protein yield**.** Yield per gram of dry weight in the crude extract in dependence of the biomass concentration during cell disruption in the 350 mL chamber of the Bead Beater (Biospec).

| Gram of dry weight per litre during cell disruption | Protein yield  [mg (g dry weight)^-1^] |
| --- | --- |
| 4.5 | 252 |
| 9.0 | 238 |
| 13.5 | 224 |
| 15.8 | 210 |
| 31.5 | 170 |
| 80.0 | 70 |

Supplementary Figure 2.2 Protein yield from disruption of hyphae. The standard concentration of bio-mass during cell disruption was 12 gram of dry weight per litre or 2 gram per disruption passage.

**Losses during purification**

To evaluate losses of protein and vanadate sensitive ATPase activity during the purification of plasma membranes these two parameters were determined in all fractions. Attention! These values are not from chemostat mycelia but from batch mycelia growing exponentially at µ_max_. It is obvious that with the discarded interphase of the two phase system about 80 % of the plasma membranes were lost thus indicating the direction for further optimization.

Losses due to discarded interface reported in the literature:

50 % (Müller *et al.* 2001; *Penicillium ochrochloron,* glass bead mill disruption)

20 % (Ugalde *et al.* 1992; *Penicillium cyclopium,* Potter disruption)

Supplementary Table 2.5 Losses during purification of plasma membranes

|  | Total protein  [mg] | | | Total vanadate sensitive ATPase activity [nmol PO_4_ min^-1^] | | |
| --- | --- | --- | --- | --- | --- | --- |
|  | Supernatant | Pellet | Sum | Supernatant | Pellet | Sum |
| Filtrated homogenate |  |  | 669 |  |  | 3407 |
|  |  |  |  |  |  |  |
| After 5.000 g, 5 min | 648 | 20 | 668 | 2943 | 372 | 3315 |
| After 10.000 g, 10 min. of supernatant 5000 g | 608 | 20 | 628 | 2107 | 740 | 2847 |
| After 100.000 g, 30 min. of supernatant 10.000 g | 492 | 55 | 547 | 0 | 1777 | 1777 |
|  |  |  |  |  |  |  |
| Loaded via MF onto the 2 phase system |  | 55 |  |  | 1777 |  |
|  |  |  | % |  |  | % |
| Upper phase (Polyethylene glycol) |  | 1 | 2 |  | 201 | 11 |
| Interphase |  | 45 | 83 |  | 1382 | 78 |
| Lower phase (Dextran) |  | 8 | 15 |  | 68 | 4 |
| **Sum** |  | 54 | 100 |  | 1651 | 93 |

**Protein determination**

Colour reagent for protein determination: For the colour reagent stock solution 50 mL of 85 % ortho-phosphoric acid were mixed with 15 mL of absolute ethanol and 50 mg Serva Blue G (concentration 0.067 % w/v), the solution was stored in a brown glass flask at 4 °C. To obtain the working solution of the reagent the stock solution was diluted 1:8 with deionized water and filtered through two pleated filters. The working solution was also stored at 4 °C but always had to be filtered right before use.

1 % (w/v) BSA stock solution: 100 mg of bovine serum albumin were dissolved in 10 mL of deionized water (concentration 10 mg/mL). Aliquots of 1 mL were frozen and stored at – 20 °C.

1 % (w/v) Triton X-100 stock solution: 0.1 g of Triton X-100 was dissolved in 10 mL of deionized water. Aliquots of 1 mL were frozen and stored at – 20 °C.

The calibration curve was determined with three bovine serum albumin (BSA) standards (stock solution 1 % w/v = 10 mg/mL; working solution 0.1 %; standard end concentrations 0.1, 0.2, 0.3 mg/mL protein). The microsomal fraction samples were diluted 1:10 with deionized water, the protein concentrations of the crude extract, plasma and intracellular membrane homogenates (all dissolved in RM 2) were determined in undiluted samples.

Protein determination was conducted in plastic cuvettes. Each was filled with 50 µL of 0.125 % w/v Triton X-100 (stock solution diluted 1:8 with deionized water), 50 µL of deionized water (reference sample)/standard/(un)diluted sample and 900 µL of colour reagent.

After a 5-minute incubation period the samples were measured at a wavelength of 595 nm (Ultrospec 2000, Pharmacia Biotech Ltd., Cambridge, England) against the reference sample with deionized water.

When conducted in micro well plates 12.5 µL of Triton X-100 (0.125 % w/v), 12.5 µL of sample and 225 µL of colour reagent were used.

For the detection of protein loss during the purification process supernatant samples were diluted 1:10 with deionized water, pellet samples 1:2. Original crude extract and microsomal fraction samples (dissolved in HM and RM 1) were diluted 1:50. Protein concentrations in the final samples (dissolved in RM 2) were measured in undiluted aliquots.

Following a 5-minute incubation period, optical density at 595 nm was determined by use of a plate reader (Sunrise, Tecan Group AG, Maennedorf, Switzerland).

**Vanadate sensitive ATPase**

For the determination of the vanadate sensitive ATPase activity the following assay parameters were varied or optimized, respectively:

For the enzymatic reaction:

- Amount of membrane protein
- Incubation time of membranes with ATP
- pH (confirming that the vanadate sensitive ATPase activity had a pH optimum of 6)
- Vanadate concentration (specific inhibitor of the P type plasma membrane H^+^-ATPase
- Triton X 100 concentration

For quantification of phosphate liberated enzymatically from ATP:

- Phosphate determination was done in principle according to Lanzetta et al. 1979 as adapted to be carried out in microtiter plates.
- Incubation time until the A630 was stable
- Linear range of the calibration curve
- Concentration of citrate added to stop further development of colour
- Time of addition of citrate
- Phosphate contaminations in the reagents

Optimized assay

Two micro well plates were used for measuring vanadate-sensitive H^+^-ATPase activity in four samples. The wells were loaded with 60 µL of tubes A to F, 30 or 60 µL of sucrose (330 mM) and 30 µL of the different samples containing 1 µg of membrane protein (diluted with precooled sucrose). Aliquots of 120 µL of deionized water (reference sample) and five standards (µM: 5.4, 10.8, 26.9, 53.9, 107.7) were pipetted into wells H 7 through H 12. The table below depicts the composition in the wells. The last component to be added was the 4 mM ATP working solution, right afterwards the plates were shaken for 20 seconds in the plate reader (Sunrise, Tecan Group AG, Maennedorf, Switzerland) using a predefined method in the respective software programme (Tecan, Magellan 6).

After a 30-minute incubation period at room temperature 120 µL of reagent C were added to the wells to start the colour reaction. The reaction was stopped after 60 seconds with 25 µL of 34 % (w/v) citrate. To guarantee adequate mixing of the contents in the wells an adhesive film was put on the plate and the plate was shaken on a vortexer for a few seconds. The colour intensity was measured in the plate reader at a wavelength of 630 nm.

Supplementary Table 2.6 Pipetting schema for the vanadate-sensitive H^+^-ATPase assay in micro well plate (96 wells). Two plates were used for measuring four samples (plate 1 with tubes A, B, C; plate 2 with tubes D, E, F). Wells H7 through H12 contain the phosphate standards; the numbers represent the respective concentrations in

µM. Other concentrations: sucrose – 330 mM; ATP – 4 mM; samples 1-4 – 33 µg/mL.

|  | **1** | **2** | **3** | **4** | **5** | **6** | **7** | **8** | **9** | **10** | **11** | **12** |
| --- | --- | --- | --- | --- | --- | --- | --- | --- | --- | --- | --- | --- |
| **A** | 60 µL tube A/D  60 µL sucrose | | | 60 µL tube A/D | | | 60 µL tube A/D | | | 60 µL tube A/D | | |
|  |  |  |  | 30 µL sucrose | | | 30 µL sample 1 | | | 30 µL sample 1 | | |
|  |  |  |  | 30 µL ATP | | | 30 µL sucrose | | | 30 µL ATP | | |
| **B** | 60 µL tube B/E  60 µL sucrose | | | 60 µL tube B/E | | | 60 µL tube B/E | | | 60 µL tube B/E | | |
|  |  |  |  | 30 µL sucrose | | | 30 µL sample 1 | | | 30 µL sample 1 | | |
|  |  |  |  | 30 µL ATP | | | 30 µL sucrose | | | 30 µL ATP | | |
| **C** | 60 µL tube C/F  60 µL sucrose | | | 60 µL tube C/F | | | 60 µL tube C/F | | | 60 µL tube C/F | | |
|  |  |  |  | 30 µL sucrose | | | 30 µL sample 1 | | | 30 µL sample 1 | | |
|  |  |  |  | 30 µL ATP | | | 30 µL sucrose | | | 30 µL ATP | | |
| **D** | 60 µL tube A/D | | | 60 µL tube A/D | | | 60 µL tube A/D | | | 60 µL tube A/D | | |
|  | 30 µL sample 2 | | | 30 µL sample 2 | | | 30 µL sample 3 | | | 30 µL sample 3 | | |
|  | 30 µL sucrose | | | 30 µL ATP | | | 30 µL sucrose | | | 30 µL ATP | | |
| **E** | 60 µL tube B/E | | | 60 µL tube B/E | | | 60 µL tube B/E | | | 60 µL tube B/E | | |
|  | 30 µL sample 2 | | | 30 µL sample 2 | | | 30 µL sample 3 | | | 30 µL sample 3 | | |
|  | 30 µL sucrose | | | 30 µL ATP | | | 30 µL sucrose | | | 30 µL ATP | | |
| **F** | 60 µL tube C/F | | | 60 µL tube C/F | | | 60 µL tube C/F | | | 60 µL tube C/F | | |
|  | 30 µL sample 2 | | | 30 µL sample 2 | | | 30 µL sample 3 | | | 30 µL sample 3 | | |
|  | 30 µL sucrose | | | 30 µL ATP | | | 30 µL sucrose | | | 30 µL ATP | | |
| **G** | 60 µL tube A/D | | | 60 µL tube A/D | | | 60 µL tube C/F | | | 60 µL tube C/F | | |
|  | 30 µL sample 4 | | | 30 µL sample 4 | | | 30 µL sample 4 | | | 30 µL sample 4 | | |
|  | 30 µL sucrose | | | 30 µL ATP | | | 30 µL sucrose | | | 30 µL ATP | | |
| **H** | 60 µL tube B/E | | | 60 µL tube B/E | | | 0 | 5,4 | 10,8 | 26,9 | 53,9 | 107,7 |
|  | 30 µL sample 4 | | | 30 µL sample 4 | | |  |  |  |  |  |  |
|  | 30 µL sucrose | | | 30 µL ATP | | |  |  |  |  |  |  |

**Reagents**

Basic reaction solution (BRL): The BRL consisted of 50 mM MES, 330 mM sucrose, 50 mM KNO_3_, 5 mM NaN_3_, 5 mM Na_2_MoO_4_*2 H_2_O, 0.1 mM EDTA (Titriplex III). All components were united in a volumetric flask (100 mL) and dissolved in 90 mL of deionized water, pH was set to 6.0 using NaOH (5 and 10 M) and the volume of the solution filled to 100 mL.

120 mM magnesium sulphate solution: 0.296 g of MgSO_4_*7 H_2_O were dissolved in 10 mL of deionized water, frozen and stored at – 20 °C.

8 mM Na-orthovanadate stock solution: 0.07256 g of Na_3_VO_4_ were dissolved in 30 mL of deionized water and stirred until the solution was clear. It was then heated for 15 minutes at a temperature of 98 °C. After cooling down the volume of the stock solution was set to 50 mL in a respective volumetric flask.

2 % (w/v) Triton X-100 solution: 0.2 g of Triton X-100 was dissolved in 10 mL of deionized water. The solution was frozen and stored at – 20 °C.

The following table depicts the composition of the six different tubes. All specifications are for a total volume of 10 mL.

Supplementary Table 2.7 Composition of the reaction solutions in tubes A through F used in the vanadate-sensitive H^+^-ATPase assay. Specifications are for a volume of 10 mL per tube.

|  | **A** | **B** | **C** | **D** | **E** | **F** |
| --- | --- | --- | --- | --- | --- | --- |
| BRL | 5 mL | 5 mL | 5 mL | 5 mL | 5 mL | 5 mL |
| 2 % Triton X-100 |  |  |  | 0.2 mL | 0.2 mL | 0.2 mL |
| 120 mM MgSO_4_*7 H_2_O |  | 0.5 mL | 0.5 mL |  | 0.5 mL | 0.5 mL |
| 8 mM Na-orthovanadate |  |  | 0.5 mL |  |  | 0.5 mL |
| Deionized water | 5 mL | 4.5 mL | 4 mL | 4.8 mL | 4.3 mL | 3.8 mL |

Tube A: -Triton/-VO_4_/-Mg Tube B: -Triton/-VO_4_/+Mg

Tube C: -Triton/+VO_4_/+Mg Tube D: +Triton/-VO_4_/-Mg

Tube E: +Triton/-VO_4_/+Mg Tube F: +Triton/+VO_4_/+Mg

330 mM sucrose solution: 11.296 g of sucrose were dissolved in 100 mL of deionized water and stored at a temperature of 4 °C.

12 mM ATP stock solution: 33.1 mg of ATP disodium salt hydrate were dissolved in 5 mL of cold sucrose solution (330 mM) and put on ice.

4 mM ATP working solution: 2 mL of the 12 mM ATP stock solution were mixed with 4 mL of cold sucrose solution (330 mM) and stored on ice. Right before use the working solution was warmed up by hand for a few seconds.

Reagent  A: 0.27 g of PVA were dissolved in 90 mL of deionized water and stirred for 30 minutes at a temperature of 80 °C. After cooling down 0.05 g of malachite green oxalate were added and the solution stirred for 60 minutes at room temperature. Reagent A was then brought up to a volume of 100 mL and stored in a brown-glass bottle at 4 °C.

Reagent B: 16 mL of 37 % HCl and 2 g of ammonium molybdate 4H_2_O were added to 20 mL of deionized water and the solution filled up to a volume of 50 mL. It was stored at 4 °C.

Reagent C: Three parts of reagent A were mixed with one part of reagent B and slowly stirred for over 30 minutes at room temperature.

Stop solution (34 % w/v citrate solution): 34 g of tri-sodium citrate 2H_2_O were dissolved in 80 mL of deionized water. The volume of the solution was set to 100 mL and aliquots were stored at 4 °C or – 20 °C (long-term storage).

**3. Section Metabolism**

**Optimized analytical methods**


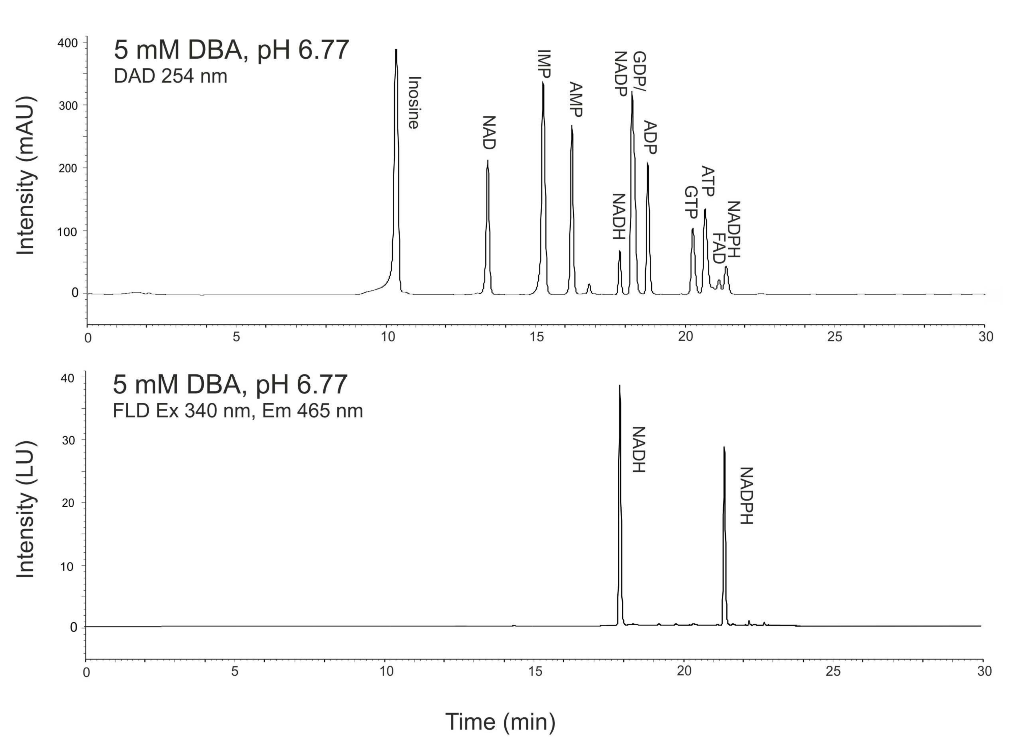


(A)

(B)

Supplementary Figure 3.1. Separation of a mixture with the 12 standard compounds Inosine, IMP, NAD, NADH, NADP, NADPH, AMP, ADP, ATP, GDP, GTP and FAD under optimized HPLC conditions recorded with DAD at 254 nm (A) and FLD (B). Stationary phase: Luna 5 µ C-8 (150 x 4.6 mm). Mobile phase: (A) 5 mM DBA in water (pH 6.8), (B) acetonitrile. Gradient: 99.5 A/0.5 B for 8 min to 99.5 A/0.5 B, in 7 min to 95A/5B, in 5 min to 90A/10B, in 10 min to 65A/35B. Flow rate: 0.8 ml/min. Conditioning: flushing for 10 min with ACN and 0.5 % H_3_PO_4_, 70:30. 10 min equilibration with 99.5A/0.5B, temperature 16 °C. Detection: DAD 254 nm, FLD Ex 340 nm, Em 465 nm. Data from Krüger (2013).

Supplementary Table 3.1. Calibration data of the optimized HPLC method for the 12 standard compounds including regression equation, correlation coefficient, linear range, limit of detection (LOD) and limit of quantification (LOQ). Data from Krüger (2013).


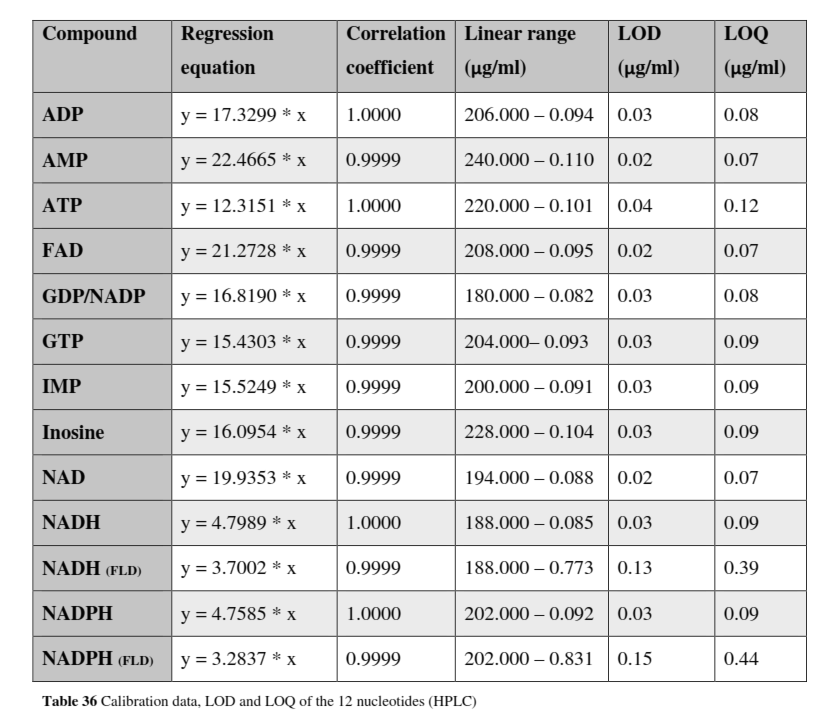


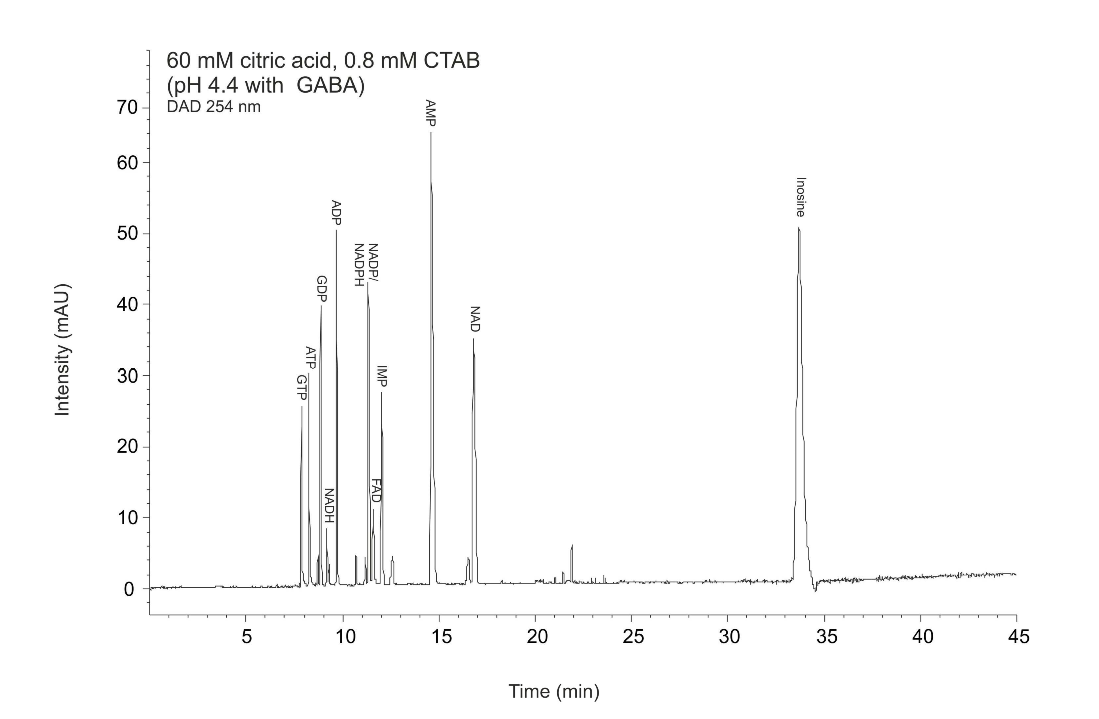
Supplementary Figure 3.2. Separation of a mixture with the 12 standard compounds Inosine, IMP, NAD, NADH, NADP, NADPH, AMP, ADP, ATP, GDP, GTP and FAD under optimized CE conditions. Capillary: Fused silica (ID 50m, effective length 62 cm). Running electrolyte: 60 mM citric acid and 0.8 mM CTAB (pH 4.2, adjusted with GABA in solid form). Temperature: 15 °C. Voltage: -25 kV. Injection: hydrodynamic, 35 mbar for 6 s. Capillary conditioning: 30 min 0.1 N NaOH, 30 min water, 15 min running electrolyte. Equilibration: 3 min 0.1 N NaOH, 3 min water, 3 min running electrolyte. Detection: UV DAD 254 nm, 360 nm. Runtime: 40 min. Data from Krüger (2013).

Supplementary Table 3.2. Calibration data of the optimized CE method for the 12 standard compounds including regression equation, correlation coefficient, linear range, limit of detection (LOD) and limit of quantification (LOQ). Data from Krüger (2013).


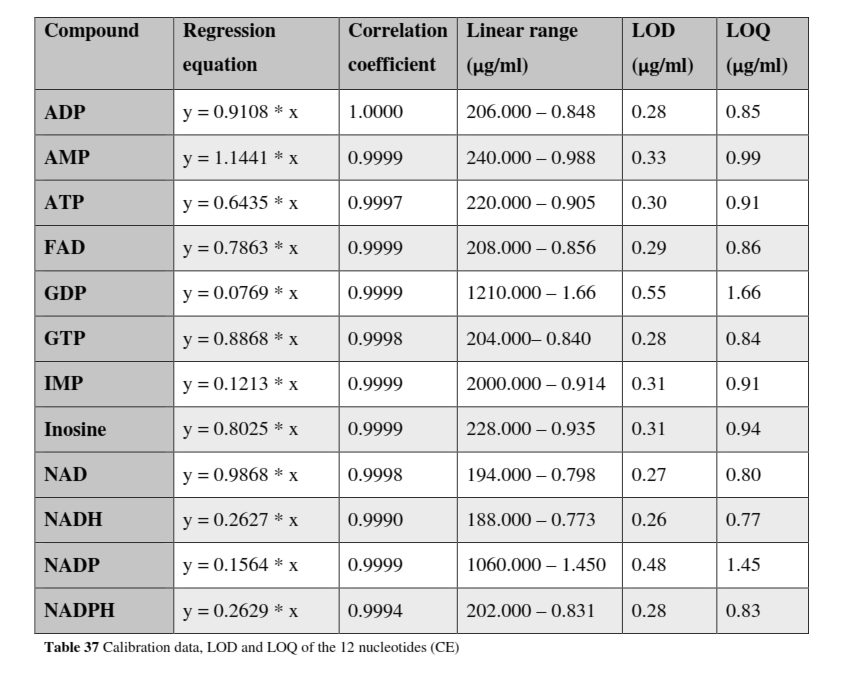


**Recovery rates**


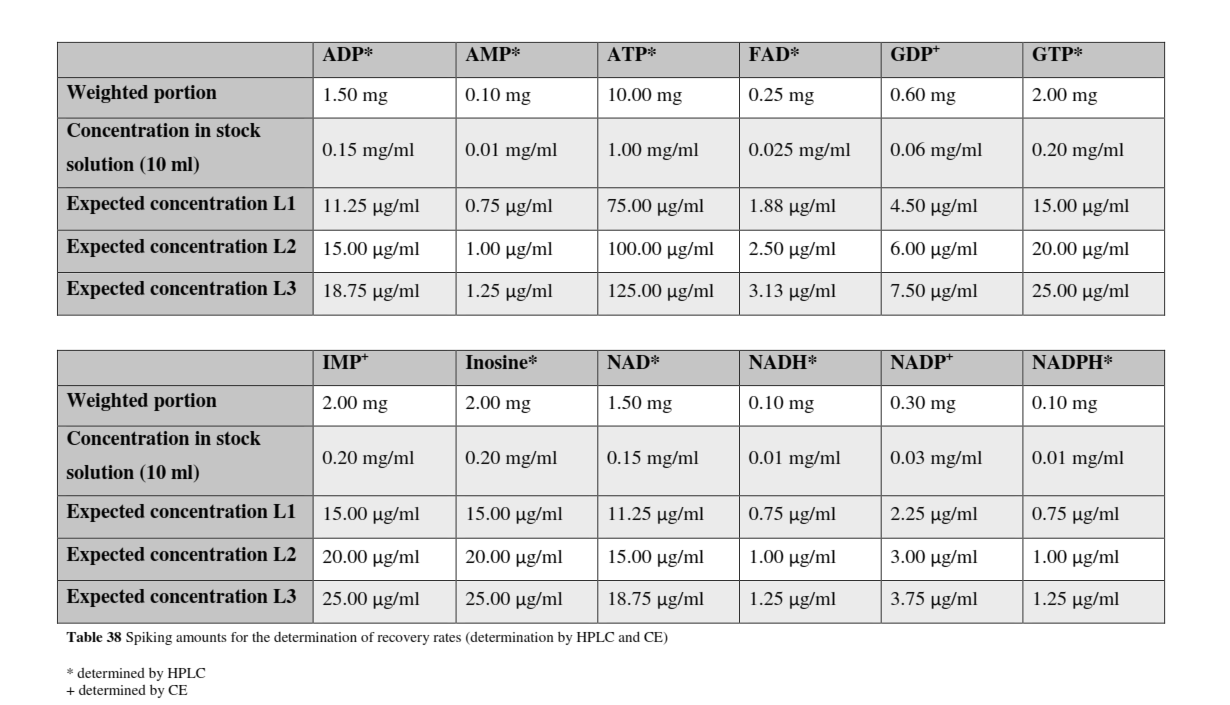
Supplementary Table 3.3. Spiking amounts for the determination of recovery rates (determination HPLC and CE). L1 … low spike, L2 … medium spike, L3 high spike. * determined by HPLC; + determined by CE. Data from Krüger (2013).


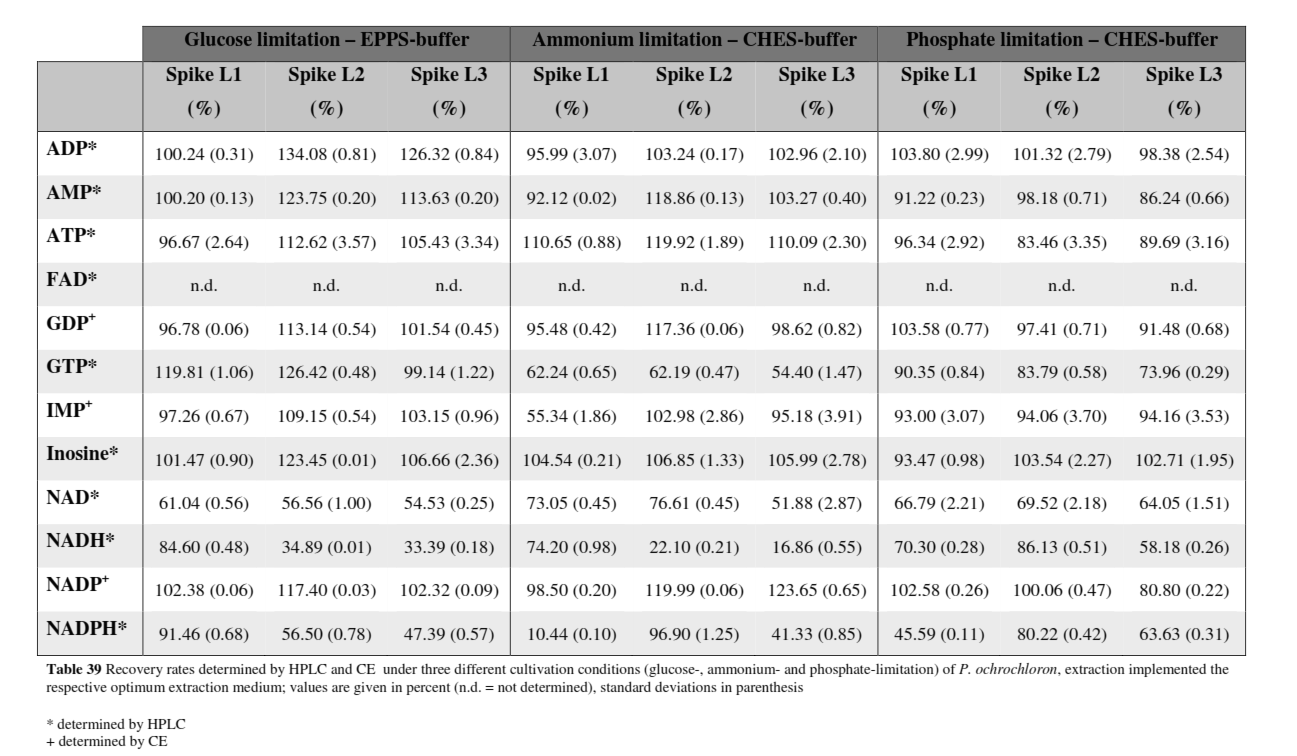
Supplementary Table 3.4. Recovery rates determined by HPLC and CE for three different nutrient limited grown *P. ochrochloron* (glucose-, ammonium- and phosphate-limitation) cultures. Extraction implemented the respective optimum extraction medium. Values are given in percent, standard deviation in parenthesis. n.d. … not determined, L1 … low spike, L2 … medium spike, L3 high spike, * determined by HPLC; + determined by CE. Data from Krüger (2013).

**Inter- and intra-day precision**

Supplementary Table 3.5. Intra- and inter-day precision of samples derived from glucose-limited chemostat conditions with EPPS-buffer as extraction solvent. Values given in percent (RSD%) based on peak area: Intra-day precision of at least n = 4, inter-day precision n = 14. n.d. … not determined; * determined by HPLC; ^+^ determined by CE. Data from Krüger (2013).


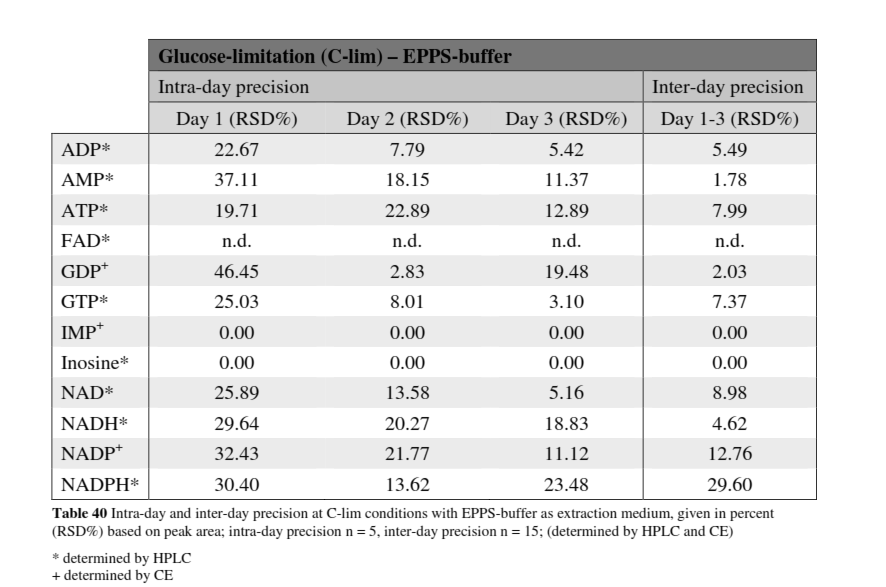


Supplementary Table 3.6. Intra- and inter-day precision of samples derived from ammonium-limited chemostat conditions with CHES-buffer as extraction solvent. Values given in percent (RSD %) based on peak area: Intra-day precision of at least n = 4, inter-day precision n = 13. n.d. … not determined; * determined by HPLC; ^+^ determined by CE. Data from Krüger (2013).


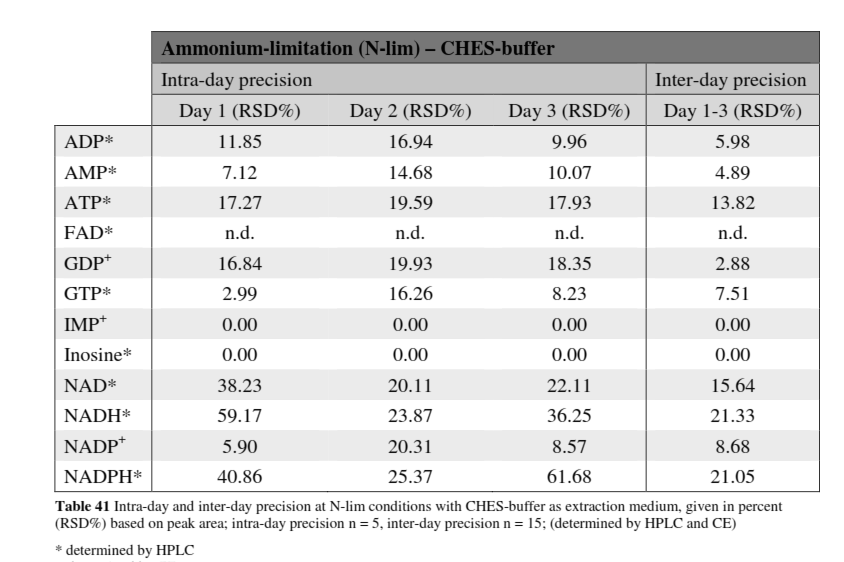


Supplementary Table 3.7. Intra- and inter-day precision of samples derived from phosphate-limited chemostat conditions with CHES-buffer as extraction solvent. Values given in percent (RSD%) based on peak area: Intra-day precision of at least n = 3, inter-day precision n = 11. n.d. … not determined; * determined by HPLC; ^+^ determined by CE. Data modified from Krüger (2013).


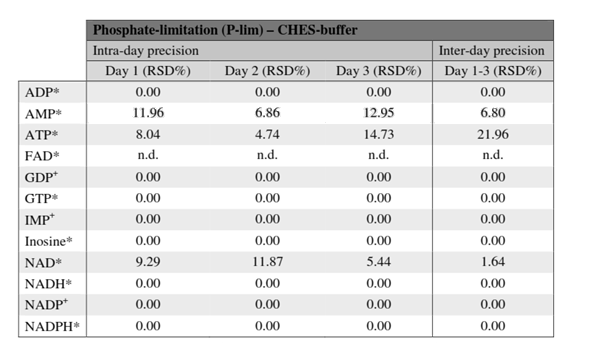


**Sample chromatograms and electropherograms**


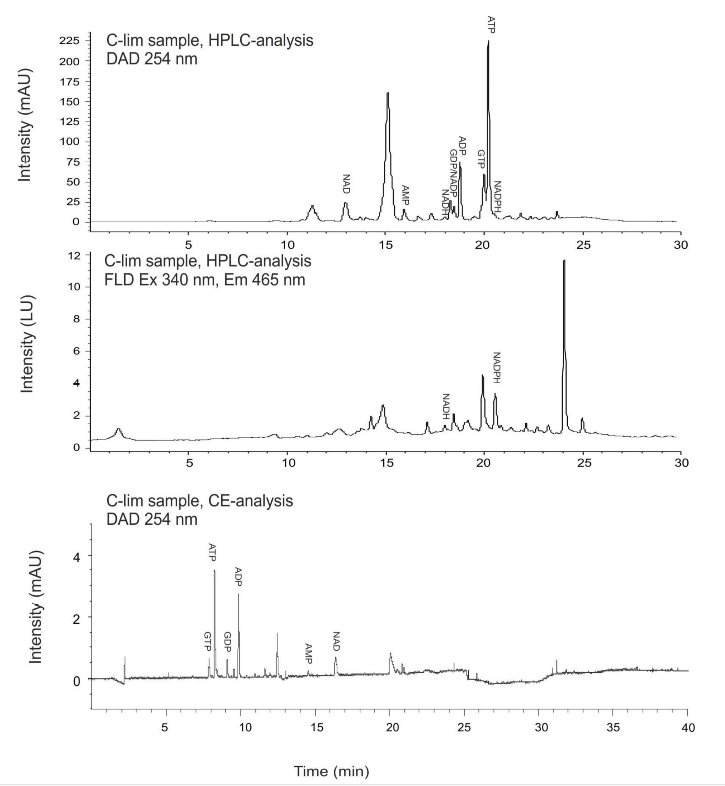


Supplementary Figure 3.3. Chromatogramm (blank substracted) and electropherogram of a typical sample of glucose-limited grown chemostat mycelium of *P. ochrochloron* CBS 123.824. The sample was extracted with EPPS-buffer, analyzed with the optimized HPLC-method (Supplementary Figure 3.1) and CE method (Supplementary Figure 3.2). Data from Krüger (2013).


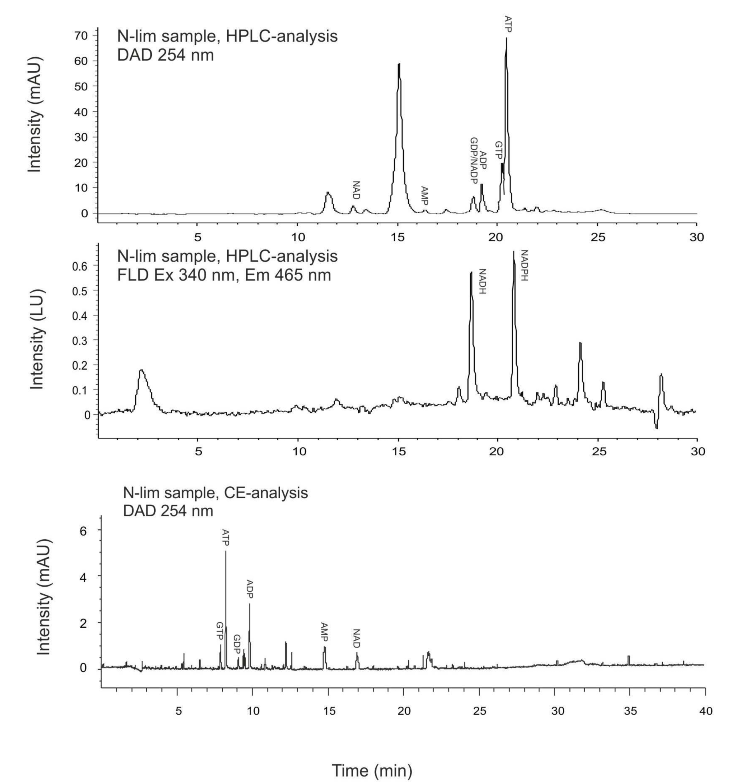


Supplementary Figure 3.4. Chromatogramm (blank substracted) and electropherogram of a typical sample of ammonium-limited grown chemostat mycelium of *P. ochrochloron* CBS 123.824. Sample was extracted with CHES-buffer, analyzed with the optimized HPLC-method (Supplementary Fig 3.1) and CE method (Supplementary Fig 3.2). Data from Krüger (2013).


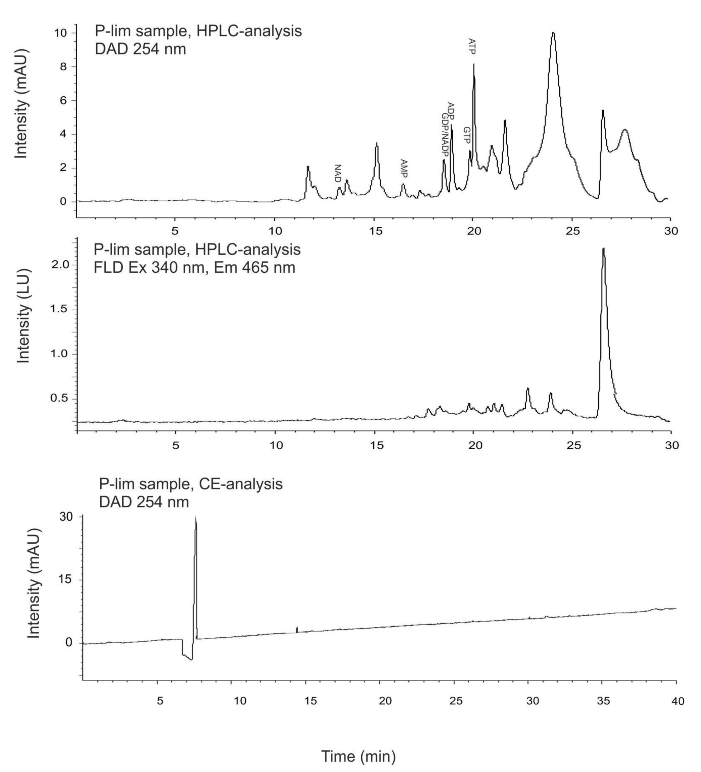


Supplementary Figure 3.5. Chromatogramm (blank substracted) and electropherogram of a typical sample of phosphate-limited grown chemostat mycelium of *P. ochrochloron* CBS 123.824. Sample was extracted with CHES-buffer, analyzed with the optimized HPLC-method (Supplementary Figure 3.1) and CE method (Supplementary Figure 3.2). Data from Krüger (2013).
